# Analytic and holistic cognitive style as a set of independent manifests: Evidence from a validation study of six measurement instruments

**DOI:** 10.1371/journal.pone.0287057

**Published:** 2023-06-13

**Authors:** David Lacko, Tomáš Prošek, Jiří Čeněk, Michaela Helísková, Pavel Ugwitz, Vojtěch Svoboda, Peter Počaji, Matěj Vais, Helena Halířová, Vojtěch Juřík, Čeněk Šašinka

**Affiliations:** 1 Department of Psychology, Faculty of Arts, Masaryk University, Brno, Czech Republic; 2 Department of Social Studies, Faculty of Regional Development and International Studies, Mendel University in Brno, Brno, Czech Republic; 3 Department of Information and Library Studies, Faculty of Arts, Masaryk University, Brno, Czech Republic; University of Belgrade Faculty of Philosophy: Univerzitet u Beogradu Filozofski Fakultet, SERBIA

## Abstract

Cognitive styles are commonly studied constructs in cognitive psychology. The theory of field dependence-independence was one of the most important cognitive styles. Yet in the past, its measurement had significant shortcomings in validity and reliability. The theory of analytic and holistic cognitive styles attempted to extend this theory and overcome its shortcomings. Unfortunately, the psychometric properties of its measurement methods were not properly verified. Furthermore, new statistical approaches, such as analysis of reaction times, have been overlooked by current research. The aim of this pre-registered study was to verify the psychometric properties (i.e., factor structure, split-half reliability, test-retest reliability, discriminant validity with intelligence and personality, and divergent, concurrent and predictive validity) of several methods routinely applied in the field. We developed/adapted six methods based on self-report questionnaires, rod-and-frame principles, embedded figures, and hierarchical figures. The analysis was conducted on 392 Czech participants, with two data collection waves. The results indicate that the use of methods based on the rod-and-frame principle may be unreliable, demonstrating no absence of association with intelligence. The use of embedded and hierarchical figures is recommended. The self-report questionnaire used in this study showed an unsatisfactory factor structure and also cannot be recommended without futher validation on independent samples. The findings also did not correspond with the original two-dimensional theory.

## Introduction

The term *cognitive style* refers to stable attitudes, preferences, or habitual strategies which determine an individual’s mode of perceiving, remembering, thinking, learning, and problem-solving [1, 2] and allow adaptation to the external world developing through interaction with the surrounding environment [3, 4]. It also represents the missing link between cognition and personality [5, 6]. The main characteristics of the cognitive style are therefore the relative stability of the construct and the absence of associations with cognitive abilities and personality [1, 2].

During the second half of the last century, dozens of models of cognitive style were created [for a review, see 1, 3, 4, 6, 7]. The majority of these works coincide in that one of the fundamental and superordinate orthogonal dimensions is the wholistic-analytic dimension we investigate in this article. According to these models, persons with a stronger preference for a wholistic cognitive style tend to process information as an integrated whole, whereas persons with strong preference for an analytic cognitive style proceed in discrete parts of that whole. In the prior research, these two types of cognitive styles were usually perceived as two polar ends of one continuum (i.e., unidimensional structure).

Today, this construct plays a key role in cross-cultural research [e.g., 8–10] but it is applied also in research on consumer behaviour [11], marketing [12], creativity [13], brain responses [14], perception of risk [15], information sciences [16] or even donation decisions during COVID-19 pandemic [17].

### Old controversy

The most representative and frequently applied theory from the wholistic-analytic cognitive style family is the field independence-dependence cognitive style (FDI), based on Witkin’s theory of psychological differentiation [2] and cognitive restructuring [18].

Throughout the theory’s history, two generations of instruments for measuring cognitive style have been introduced: maximum performance tests (1^st^ generation) and self-report questionnaires (2^nd^ generation). According to some critics, the first generation of methods assessed cognitive ability rather than cognitive style since unsuitably high associations of cognitive style with general intelligence, spatial ability, working memory, attention, and academic achievement have been found in previous studies [e.g., 19–24].

Concerning second generation methods, some authors discovered high associations with personality traits questioning the validity [e.g., 20, 25, 26]. Self-report methods are also often unrelated to performance-based measures of cognitive style, an observation which challenges their convergent validity [e.g., 20, 27, 28].

Another crucial aspect of the validity of cognitive styles is stability of the construct. Cognitive styles may shape themselves throughout a person’s life [29], be affected by various socio-cultural factors [30, 31] and be generally considered dynamic [32] and task dependent [4]. Nevertheless, they should remain relatively stable in the short to medium-term. The contemporary body of knowledge, however, remains inconclusive since some studies have found that cognitive styles are stable [e.g., 33, 34] and others have found they may change significantly, for instance after specific training [e.g., 35, 36].

Finally, some past studies criticized the generally poor or unknown psychometric properties of instruments which measure cognitive style [e.g., 37–39] and highlighted the absence of combinations of mixed-methods applied in cognitive style assessments [40].

### Current perspective

Strongly inspired by the cross-cultural differences between “modern” and “traditional” cultures found in Witkin’s FDI [see 31, 41] and between Western and Eastern countries at similar level of technological development [42], as well as by Sloman’s distinction of two cognitive systems of reasoning [43], Nisbett with colleagues proposed the new and currently predominant theory of analytic and holistic cognitive style twenty years ago (AH) [for a review, see 44–46] which should have overcome the issues of prior research. (The term *wholistic* is used more broadly and refers to holistic cognition in general, whereas *holistic* is a term used purely in Nisbett’s cross-cultural theory. In this article, we keep this distinction.) Throughout history multiple tasks of measuring AH were introduced ranging from self-report inventories and performance-based measures based on older wholistic-analytic cognitive style methods, through new computer-based instruments, to the usage of modern technologies such as virtual reality or eye-tracking. In contrast to the older methods, these methods usually incorporate specific tasks for both analytic and holistic cognition and thus demonstrate the desirable shift from unidimensional to a two-dimensional structure. We identified four main clusters of methods which attempt to eliminate the shortcomings of measurement summarized in the previous section.

### Performance-based measures based on the wholistic-analytic family

These methods stem from the early experiments of Witkin and Asch on space orientation and the FDI theory. Based on their previous work, they formulated the *Rod and Frame Test* (RFT) [18]. In the RFT method, participants are asked to set a rod, embedded in a square, to the subjective vertical position, regardless of the surrounding frame. The method is partially based on Wertheimer’s [47] tilted-mirror experiment and is derived from the little-known Gestalt principle called frame of reference. The improved version of RFT is called *Framed-Lined Test* (FLT) [48]. Compared to the RFT, where the ideal solution strategy is always field independence, the FLT takes into account both the absolute and relative fields of reference in two individual subtests. Despite its 19-year history, the psychometric properties of the FLT are unknown. Only two studies have verified its reliability via internal consistency, which was even inadequate (αs < .70) [cf. 49, 50].

The FDI is also commonly assessed via the *Embedded Figures Test* (EFT) [18]. These methods are based on *Gottschaldt’s embedded figures* [51], which are complex figures composed of simple figures. Participants are instructed to spot a simple form within a more complex figure. The psychological principle beyond these figures lies in the Gestalt principles of figure - ground organization, especially the laws of proximity, similarity, good continuation, closure and mirror symmetry [47]. Improved version of EFT is called *Cognitive Style Analysis* (CSA) [7] which contains verbal-imagery and wholistic-analytic cognitive style dimensions. Similarly to the FLT, the CSA enhances the reductionist unidimensional approach of the EFT and incorporates two subtests of AH cognitive style measurement. The CSA (unlike EFT) also does not correlate with intelligence [52], personality [53] or academic achievement [54], although some serious issues with its test-retest reliability have been revealed [e.g., 55–58]. Hence, the *Extended Cognitive Style Analysis–Wholistic/Analytic* (E-CSA-W/A) was proposed by Peterson and colleagues [57, 59]. E-CSA-W/A showed sufficient split-half reliability, parallel forms reliability and test-retest reliability [57, 60] and a lack of association with mathematical performance [61], intelligence and personality [59].

### Performance-based measures based on global-local family

The following group of methods is based on global and local processing of *Navon’s hierarchical figures* [62, 63], i.e., a large figure (global level) composed of small figures (local level). These figures were created with respect to the Gestalt principles of grouping, especially in proximity and continuity [47]. Compared to the *embedded figures* used in E-CSA-WA, *hierarchical figures* are self-contained and not nested in the surrounding context. These methods were not originally proposed for AH measurement. In fact, probably the first use of *Navon figures* in the context of cognitive styles can be traced to 2006 [64]. From the perspective of analytic and holistic cognitive styles, local processing corresponds to the analytic cognitive style and global processing to the holistic cognitive style [64]. Today, different versions of *Navon figures* are frequently used to estimate a person’s global and local processing.

These modifications differ at two levels: 1) the type of figure and 2) the aim of the task. The type of figure can be either verbal (e.g., numbers, logograms, latin script) or non-verbal (e.g., geometric shapes, abstract drawings, specific objects such faces). Both verbal and non-verbal types can be mutually combined (e.g., a large letter composed of smaller geometric shapes). Global and local features can also be combined; each figure may be congruent (local features are the same as global features) or incongruent (local features differ from global features) [62]. Distinction according to the aim of the task is less complicated since the task’s aim might be to find the correct answer (i.e., *Navon Search Task*) or to select an answer which is as similar as possible to the original figure (*Navon Similarity Matching Task*) [65].

Evidence of the psychometric properties of *Navon figures* is rather mixed. Some studies suggested that these methods have satisfactory test-retest reliability [66, 67] and split-half reliability [68, 69], while others directly questioned their validity and reliability [70, 71]. Hierarchical figures are also not associated with general intelligence [72].

### Self-report questionnaires

Despite self-report questionnaires being relatively common in the assessment of other cognitive styles, their use specifically for AH measurement remains relatively scarce. Nevertheless, a few inventories have been created exclusively for the measurement of AH as defined above. The best examples are the two-dimensional *Holism Scale* (HS) [73], and four-dimensional *Analysis-Holism Scale* (AHS) [74, 75]. AHS, the most often used questionnaire in AH research, showed discriminant validity with individualism/collectivism and independent/interdependent self-construal scales and concurrent validity in terms of weak associations with the categorization task [76]. However, its factor structure was not ideal and internal reliability was not entirely satisfactory in some subscales, and, therefore, two brief versions of AHS (AHS-12 and AHS-4) that overcome these issues were proposed [77]. Recently published four-dimensional *Holistic Cognition Scale* (HCS) [78] also demonstrated satisfactory factor structure and addressed these well-known shortcomings of AHS.

### Other methods

Methods other than those mentioned above have also been used within the AH paradigm. Quite often, these studies applied complex visual stimuli of artificially created or natural *visual scenes* as stimulus material [e.g., 79, 80]. Another frequently used set of tasks is based on the *categorization* (*triad*) *tasks* [76] and the *change blindness task* [81]. Na and colleagues [50] described other six tasks not as commonly employed as the methods described above. Generally, these tasks do not represent any formally standardized methods, and scholars usually use their own ad-hoc stimuli and interpret the validity of tasks according to the patterns of results expected in certain cultures. Their psychometric properties are therefore unknown. The only exception is research by Na and colleagues [50], who found that the internal consistency of these tasks varies significantly (αs ranged from .24 to .96) and that the test-retest reliability of four of these methods is moderate at best (*r*s ranged from .47 to .70).

### New challenges

#### Uncertain or unknown psychometric properties

Despite the relatively large body of literature which describes various AH measurement methods, evidence of psychometric properties in most of them remains unknown or ambiguous. Studies which applied self-report questionnaires and *FLT* did not report any evidence of stability in construct, and *Navon-based* methods reported unclear results. Even studies which provided some evidence of validity or reliability of measures can be disputed (e.g., *E-CSA-WA* instrument), mainly because of statistical and methodological reasons (inappropriate analyses, sample size or composition).

The most serious problem, however, is the lack of concurrent validity in AH methods. As with FDI, AH measurement shows inconsistent associations between methods. Specifically, the *FLT* only weakly associated with the *change blindness* (*r* = .19) and *causal attribution* (*r* = .22) tasks, and no associations were detected with the other nine AH measurement methods [50, 82]. *Navon hierarchical figures* also barely associated with *Gottschaldt embedded figures* [e.g., 64, 70, 72, 83], except for one study, which found a strong association [cf. 84].

Some studies reported low or no correlations between various modifications of *Navon figures* [66], thereby also challenging the convergent validity of this method. A lack of association was also observed between self-report questionnaires and performance-based measures of AH [e.g., 74, 85]. Hence, scholars in the field appear to be using methods which might not even be related to each other but are interpreting their findings as differences under the same attributes (i.e., analytic and holistic cognitive styles).

The final important factor to consider concerns self-report questionnaires. Despite the validation studies of AHS and HSC using desirable structural equation modeling techniques to verify its factor structure, only a few studies have already provided any evidence of cross-cultural comparability (as far as we know, only [77]), such as scalar measurement invariance [see 86].

#### Distribution of reaction times and their relationship to cognitive processes

Reaction times (RTs) have held a prominent position in psychological research of analytic and holistic cognitive styles. To analyse RTs, however, scientists have typically used the statistical techniques they were the most familiar with, such as analysis of variance on the sample mean [87, 88], although this was found unsuitable in many of its applications [89] because RTs are usually not identically and independently distributed *(iid*) as a result of trial-by-trial sequential effects. More importantly, in a majority of cases, RTs are not normally distributed (*Gaussian*) but rise rapidly on the left and have a long positive tail on the right [88, 89]. This feature of RTs may have produced misleading or potentially contradictory results [90].

One of the major challenges of current AH research is therefore the incorporation of suitable methods of statistical analysis of RTs. These methods would reflect not only the specific RT distribution but also its true intrinsic relationship to the cognitive construct of AH *per se*. Between the most commonly applied approaches belongs *distribution analysis*, *joint models* and *process models* [for a review, see 91, 92] which we are applying in this article. Since not a single article has yet used appropriate estimates of RTs in AH measurement, it is possible that all previous AH studies did not compare the real differences in AH cognitive traits, but rather the differences in psychomotor tempo, stimulus encoding, response carefulness [93] or working speed [94].

#### The issue of dimensionality: Futility of derived indices

Since Nisbett with his colleagues [44] introduced AH as two “systems of thought”, it is considered and especially measured as a two-dimensional construct [see also 42, 74, 76]. According to their original formulation, AH represented two separate and perhaps even qualitatively different cognitive processes that might be mutually independent and that, at the same time, might coexist simultaneously. For instance, current neuroscience evidence suggests these two processes are reflected by differences in brain activity [e.g., 14, 95, 96]. Therefore, some individuals might show a tendency to reason or perceive analytically and others holistically (naturally, some might show high or low preferences of both styles).

However, researchers often ignore this original conceptual postulation of AH theory, and reduce the amount of information in their data by treating AH as a uni-dimensional structure. This might be understandable in self-reported questionnaires due to the item wording [74], but not in performance-based methods that almost always offer two separate analytic and holistic subtests. Despite that, scholars commonly calculate derived indexes, such as difference or ratio between mean or median of RTs from both subtests, and use them as a single uni-dimensional indicator of cognitive style without any justification. For example, the manual of *E-CSA-WA* suggests calculating the main index as a ratio of median reaction times between analytic and holistic subtests [97], and *Navon-based methods* often use the difference between subtests as an indicator of the global precedence score [e.g., 98]. Even in *FLT* some scholars create derived indexes [e.g., 99]. Such an approach, however, cannot be regarded as appropriate, because it is not only rather reductionist, but in some cases, it is clearly misleading and unreliable [see 100, 101]. Because of the proposed two-dimensional nature of the AH construct, scholars are not forced to use any derived indices and instead analyse scores from multiple subtests separately.

In summary, it seems that the “old controversy” might have survived to this day, even after a face-lift in the form of analytic and holistic cognitive style theory. Many new challenges have also been raised recently. Hence, the aim in the present article is to verify the psychometric properties of methods which measure AH. To do so, we implemented several important steps: 1) application of relatively recently created methods of measuring AH to overcome the issues of the old controversy (e.g., holistic style is not perceived as inferior to analytic style); 2) the use of multiple and different methods; 3) analysis of discriminant validity with personality and intelligence; 4) verification of the stability of the construct; 5) collection of a large sample of the general population (i.e., non-student); 6) no derived indices; 7) application of advanced statistical procedures for RT estimation.

## Methods

### Analytic plan

**Fig 1:**
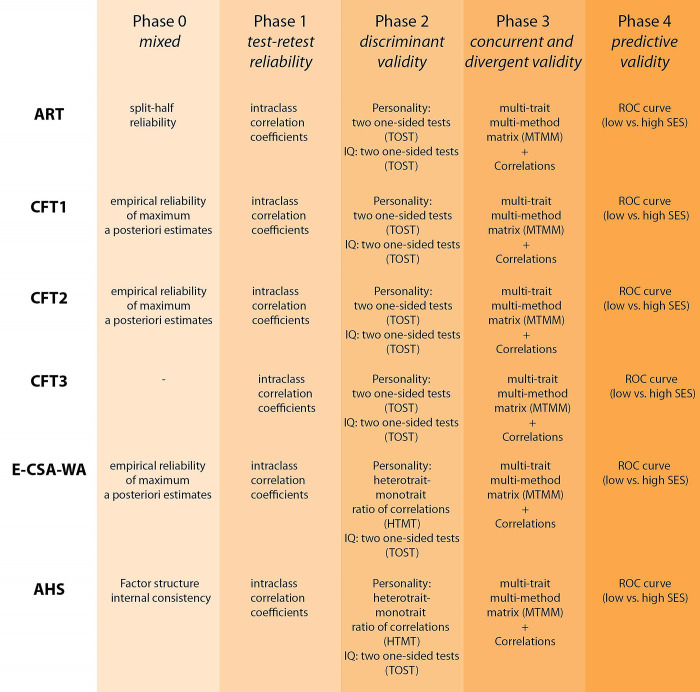
Validation process.

The hypotheses, statistical analyses and data cleaning procedures were pre-registered before data collection (see https://osf.io/w483c). The hypotheses were pre-registered as a validation process composed of five phases (Fig 1). In the first phase, specific aspects of validity and reliability (e.g., factor structure, internal consistency, split-half reliability) are verified. This phase was designated Phase 0 since it is not relevant to all the methods. In Phase 1, all methods are tested for their stability in time (test-retest reliability). In Phase 2, the discriminant validity of the methods is verified. In Phase 3, the concurrent and convergent validity of the methods is assessed. Finally, in Phase 4, predictive validity is estimated. Throughout the analysis, methods which clearly demonstrate insufficient quality do not progress to the next phase. The deviations from a pre-registered analytic plan are described in Appendix 1 in S1 File. The data, methods and *R* source codes are available online (see https://osf.io/7ezax/). The study has been approved by the Masaryk University Ethical Board (EKV-2020-118).

### Measures

#### Methods for measurement of AH

**Fig 2:**
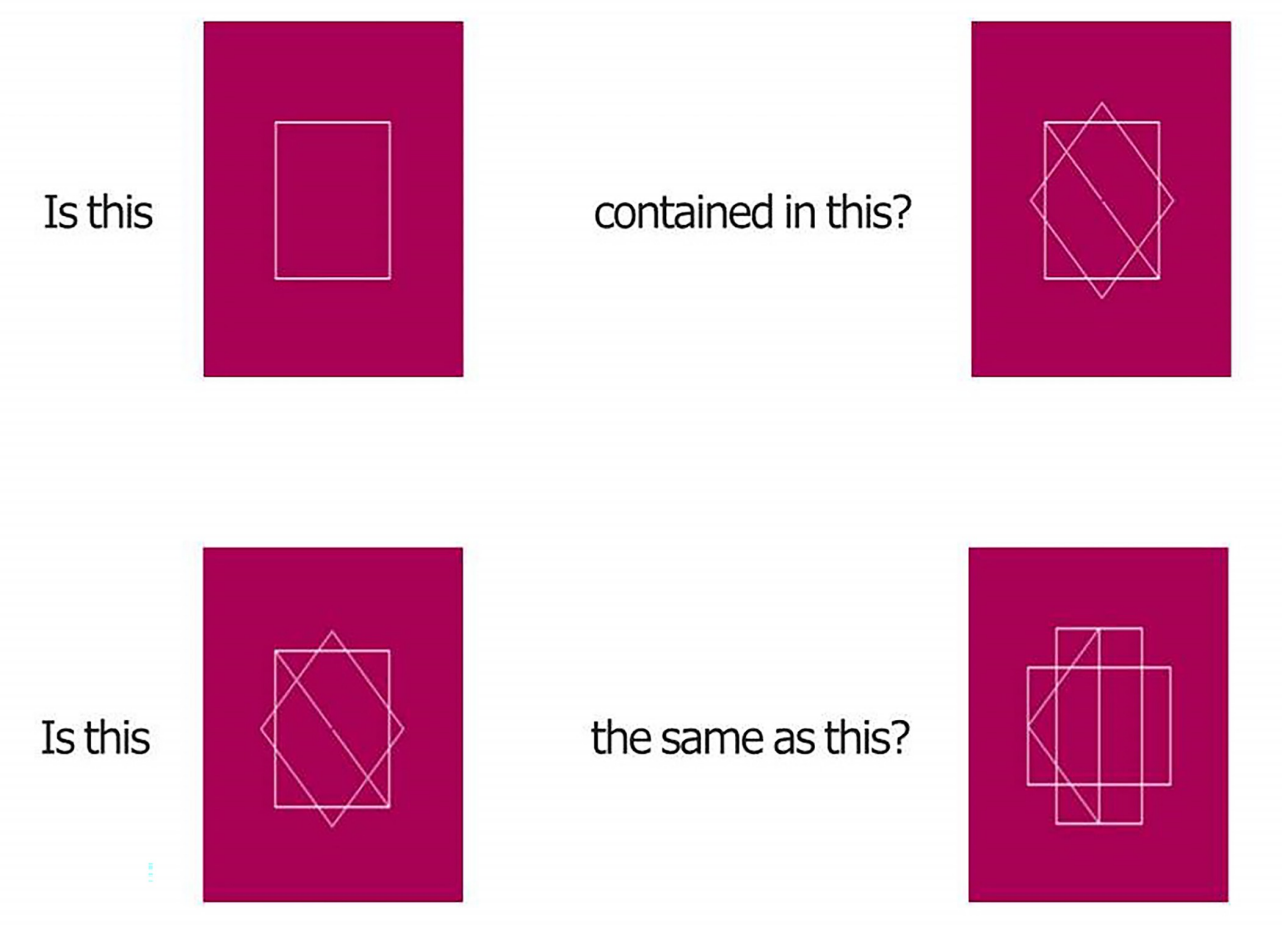
Example of E-CSA-WA stimuli.

*Extended Cognitive Style Analysis–Wholistic/Analytic (E-CSA-WA; see Fig 2) [57, 59].* It contains 80 items (40 analytic and 40 holistic). In a holistic subtest, participants are presented with two complex figures and their goal is to identify whether these figures are identical. In an analytic subtest, participants are exposed to one simple and one complex figure, and their goal is to reveal whether the complex figure contains the simple figure. Participants respond by pressing one of two keyboard buttons.

**Fig 3:**
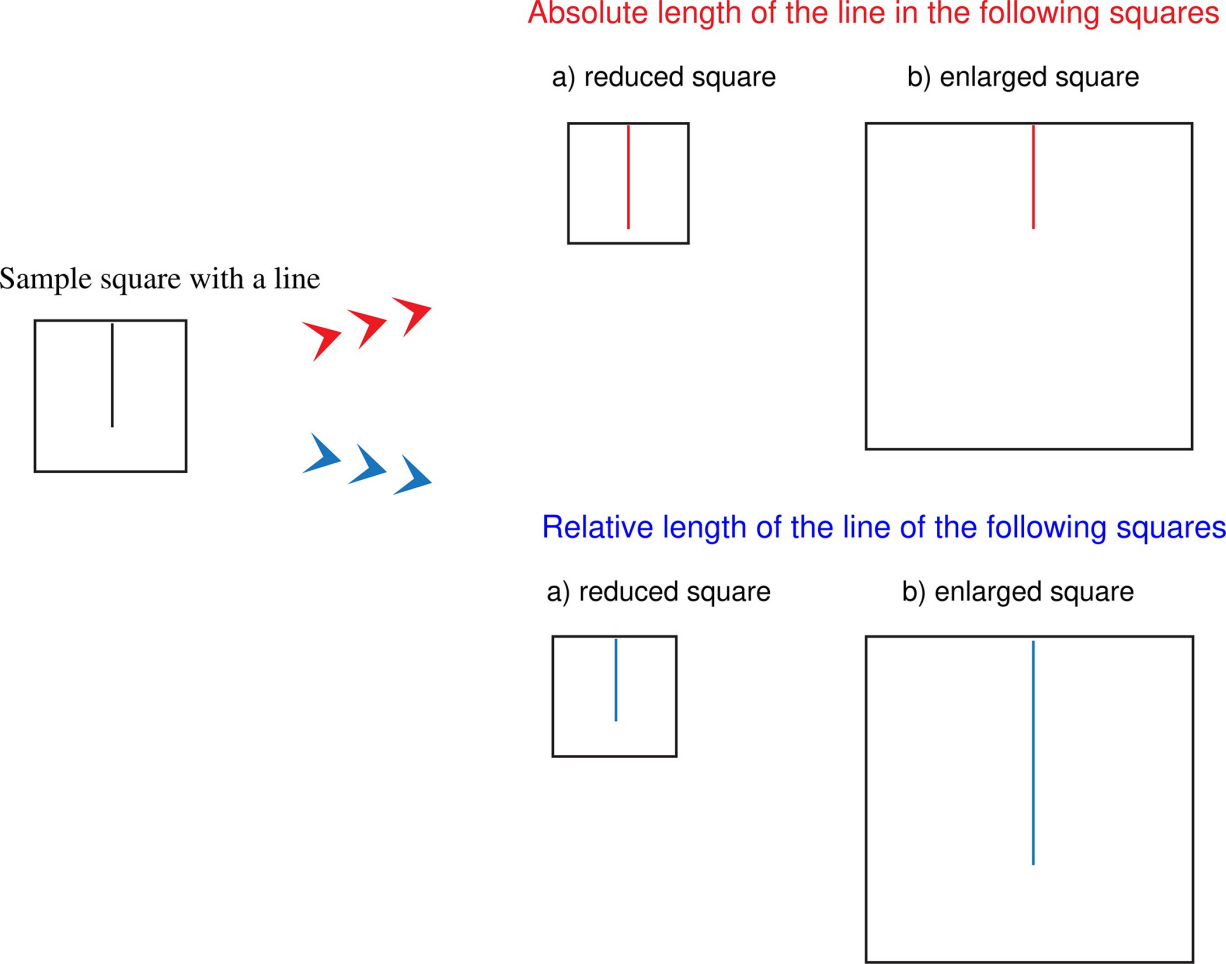
Example of ART stimuli.

*Absolute-relative test (ART)*. The ART is a computer-based adaptation of pen-and-paper *FLT* [48; line lengths were adapted from 102] Participants are exposed to the original stimulus (square with a vertical line). Their goal is to draw a line of exactly the same absolute length as the original, regardless of the size of the square (analytic task) or line that proportionally corresponds to the proportion of the line and side of the square of the original stimulus (holistic task). The original stimulus is presented for 5 s, followed by a mask presented for 100 ms. It contains 12 items (6 for analytic and 6 for holistic subtest, see Fig 3).

**Fig 4:**
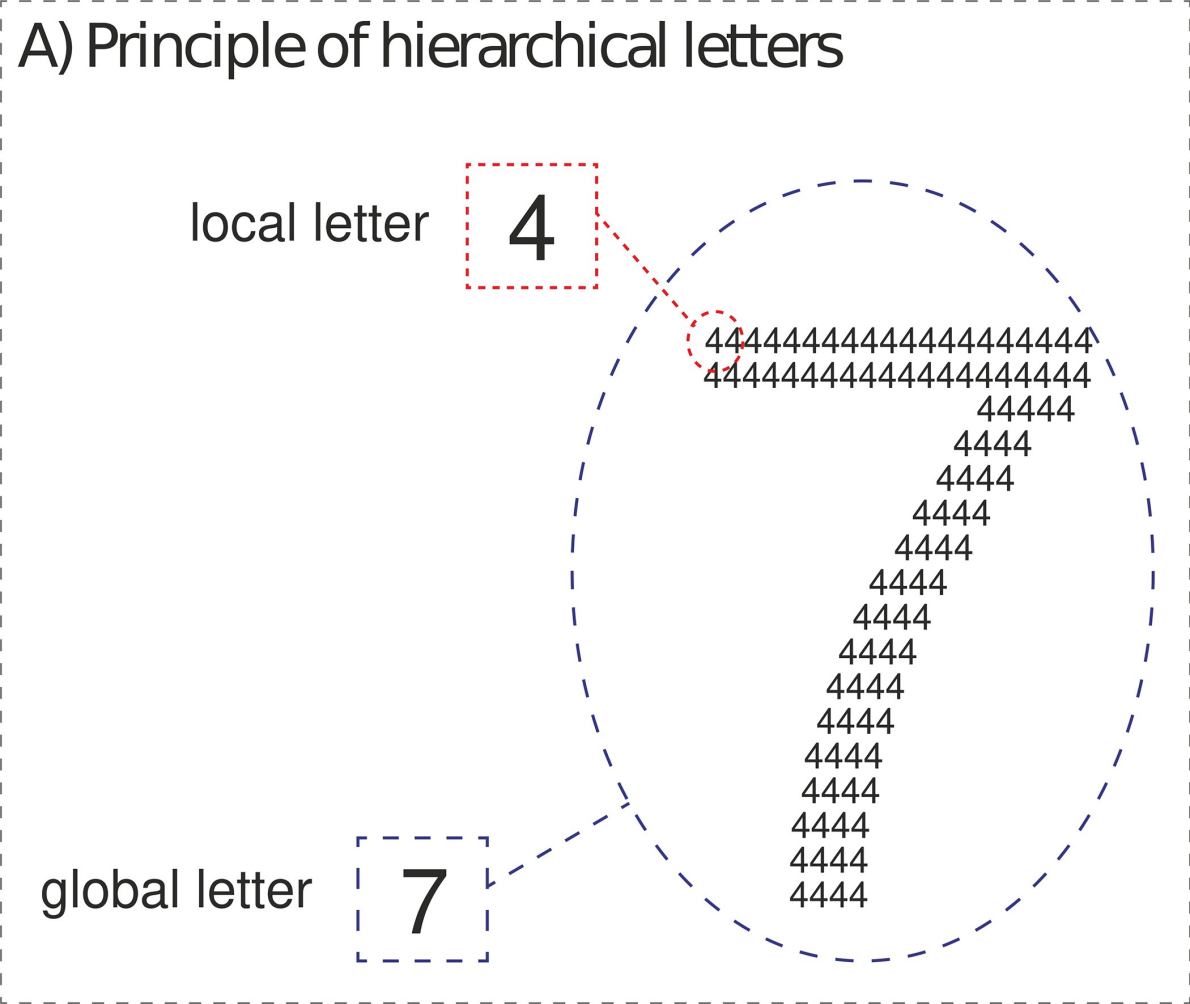
Example of CFT1 stimulus.

*Compound Figure Test 1 (CFT1) [9, 103].* CFT1 is a verbal (numbers) and incongruent form of the Navon Search task (Fig 4). Participants have to choose the correct answer from four options and indicate it with a mouse-click. The method is composed of 32 figures (16 for local identification and 16 for global identification). Fixation crosses are presented before each trial (for 500 ms) and the figure remains visible until the participant’s response.

**Fig 5:**
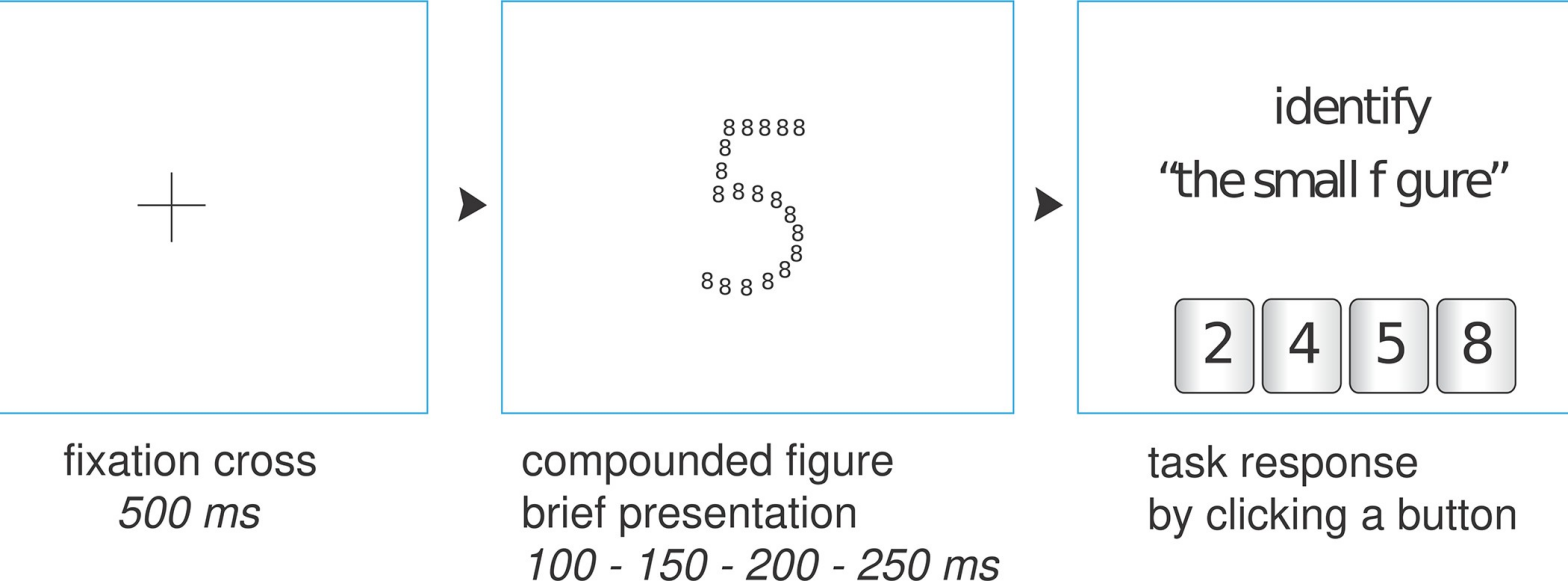
Example of CFT2 stimulus.

*Compound Figure Test 2 (CFT2)*. CFT2 is an extended version of CFT1 (80 items instead of 32) with several modification: 1) local features are larger, decreasing the advantage of the global features [104]; 2) items are block-randomized (participants do not know whether they will identify a local or global feature in the next stimulus); 3) the presentation time is shorter; original stimulus disappears (after 100, 150, 200 or 250 ms; the time is the same for each block of 20 items; see Fig 5). All other settings are the same as with CFT1. This measure was created from previous research [68, 98].

**Fig 6:**
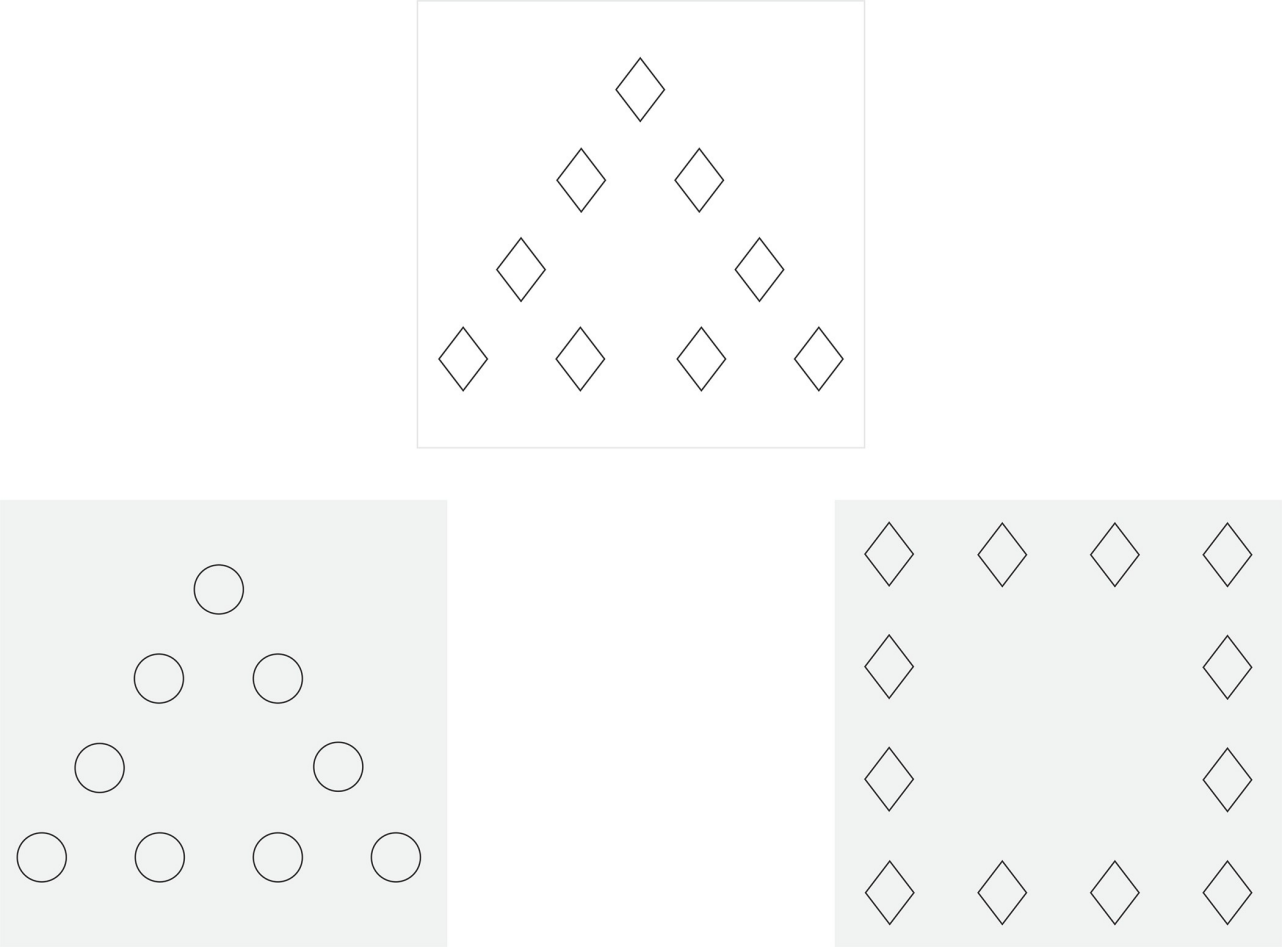
Example of CFT3 stimuli.

*Compound Figure Test 3 (CFT3)*. CFT3 is a non-verbal (geometric shapes) and incongruent form of the Navon Similarity Matching Task, which was created from previous research [65, 105, 106]. The participant does not choose the correct answer, but rather chooses a preferable answer from two options. CFT3 contains 20 items, with one sample stimulus and two options (the first shares its global feature with the original stimulus, the second shares the local features; see Fig 6). The participant is instructed to choose the option which, according to his/her opinion, is more similar to the sample stimulus.

*Analysis-Holism Scale (AHS) [74, 75].* AHS contains four subscales (locus of attention, causal theory, perception of change and attitude toward contradictions) and twenty-four 7-point Likert items (1 = strongly disagree, 7 = strongly agree) with six items per subscale.

### Methods for discriminant validity

*Big-Five Inventory 2 (BFI-2)*. The BFI-2 [107] is an updated version of BFI [108] and measures a theoretically expected five-factor model of personality (with 15 facet subscales): extraversion, agreeableness, conscientiousness, negative emotionality and open-mindedness. The BFI-2 is composed of 60 items with 5-point Likert scales (1 = disagree strongly, 5 = agree strongly). We administered the adapted and validated Czech version by Hřebíčková and colleagues [109].

**Fig 7:**
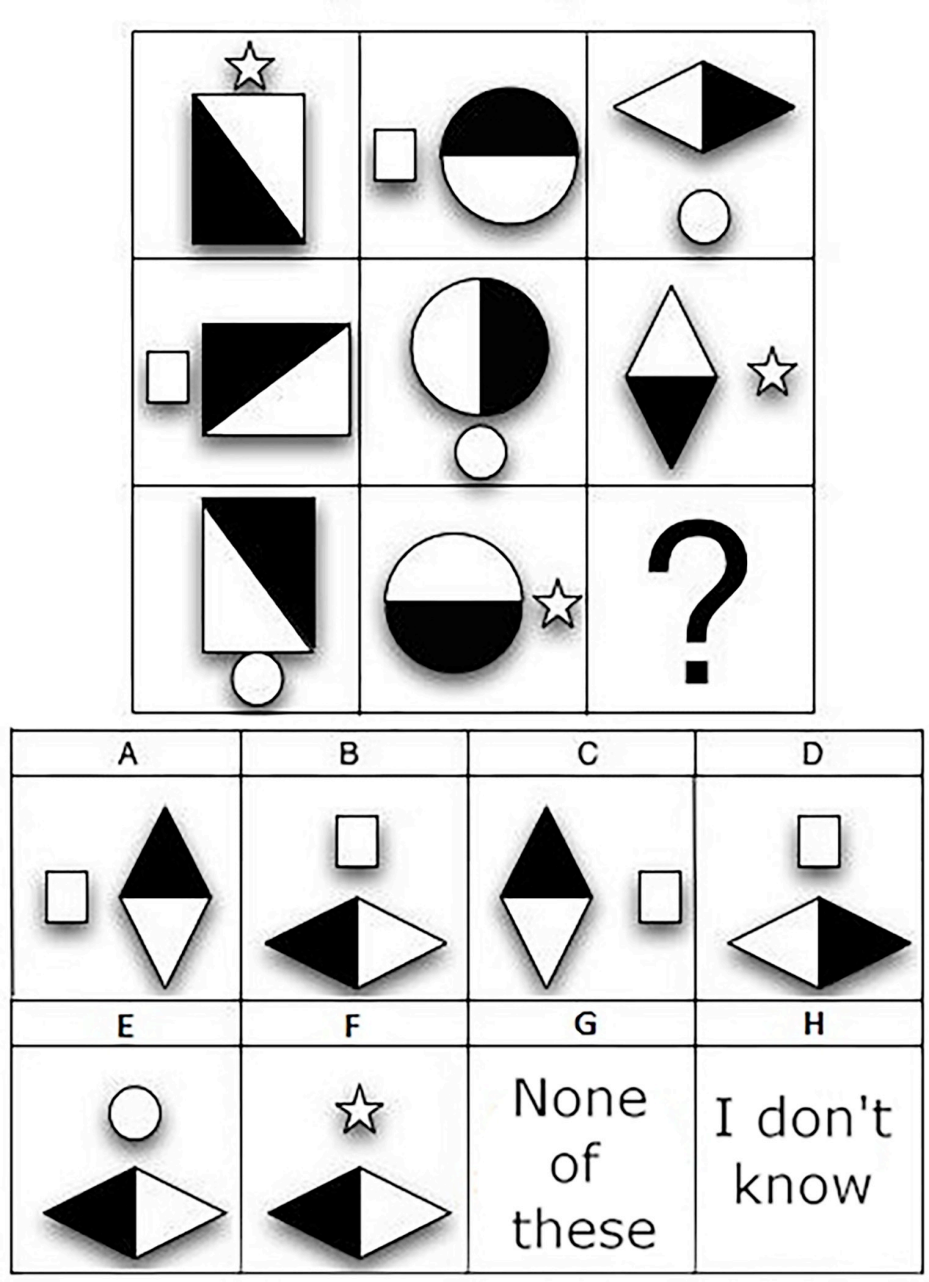
Example of an ICAR matrix reasoning subtest.

**Fig 8:**
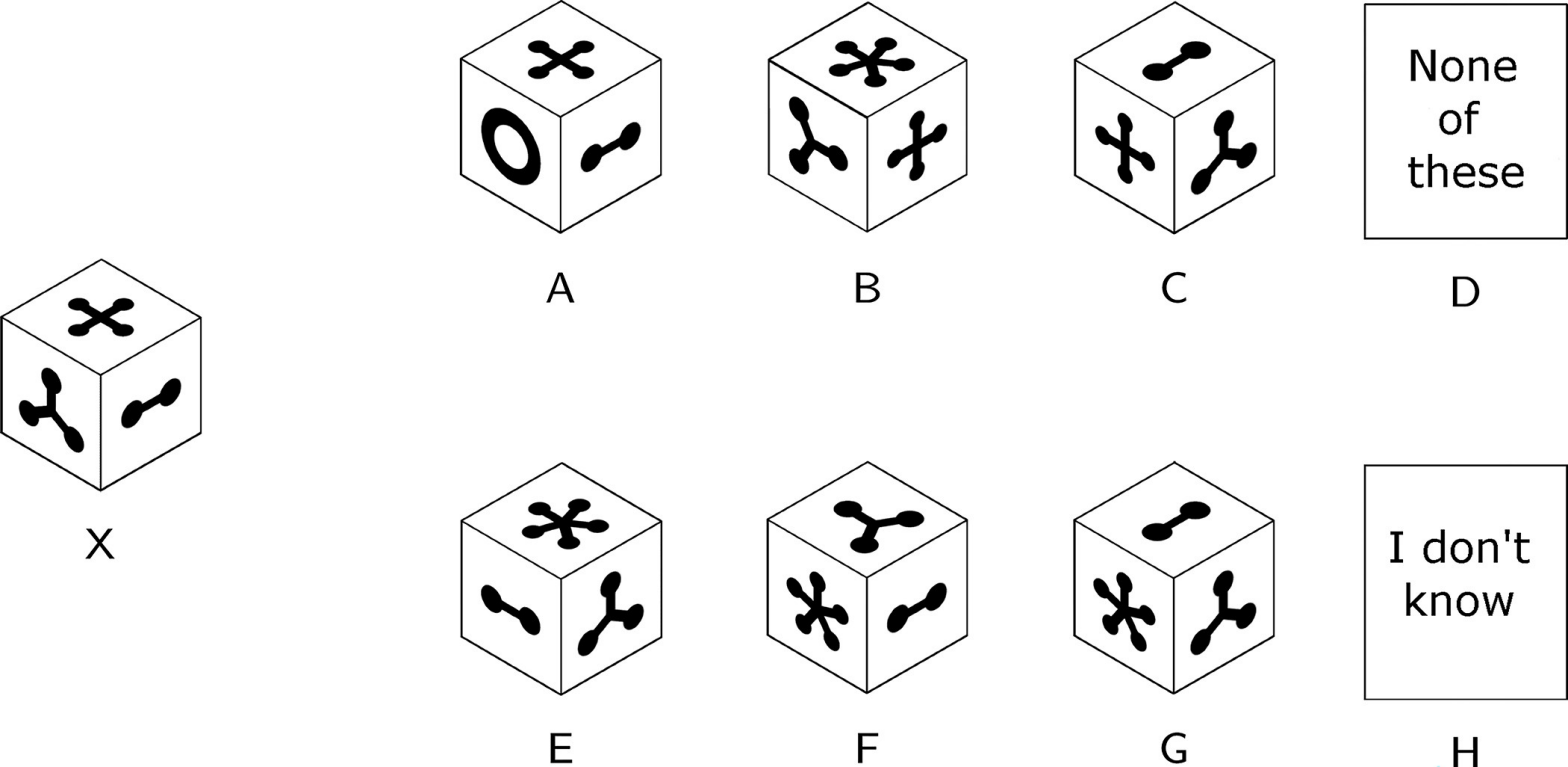
Example of an ICAR three-dimensional subtest.

*International Cognitive Ability Resource (ICAR) [110].* We used ICAR matrix reasoning (11 items, similar principle to Raven’s progressive matrices; see Fig 7), three-dimensional rotation (24 items; see Fig 8) for estimating general intelligence, and computer-generated number series subtests (11 number series, randomly selected for each item model). The ICAR generally showed satisfactory psychometric properties [111, 112].

Besides these methods, we asked participants also about their age, gender, education, martial status, number of siblings, and socio-economic status. It was measured via 4-category self-report scale (poor, lower mid, mid, upper mid).

### Procedure

#### Method development procedure

Great care was given to the process of translation to reduce potential method bias caused by potential shifts of meaning and resulting conceptual inequivalence [113, 114]. Even though the complete elimination of method bias caused by the translation might be improssible, there are methods that have proven to be effective at least in its reduction [115]. In the first step, the test methods were translated from English to target languages using a back-translation procedure by two independent translators. In the second step, the English original and the back-translation were compared by both translators. Any differences between translations were discussed and evaluated for potential shift in meaning. This step was supervised by authors of the test methods. We performed two quantitative pilot studies (*N*1 = 32, *N*2 = 21) and qualitative cognitive interviews (*N* = 7). From these three pilot studies, we clarified the instructions to reduce any potential misunderstandings. All performance-based methods contained practice trials with feedback.

#### Testing procedure

We initially created set of 48 different strata based on the combination gender, education level and age which proportionally corresponded to their representation in the general Czech population [116]. These target subgroups of the general population were then addressed in relevant groups in social network and thematic websites using the snowball method, resulting in a relatively balanced pool of participants of 600 volunteers. Forty randomly selected participants were given a reward of CZK 2,000 (approx. 40x €80).

All participants were tested online in two data collection waves. The first wave was conducted in early May, 2021, the second was collected from mid-June to mid-August, 2021. During the first wave, participants completed all methods which focused on cognitive style assessment, three subtests of ICAR and a demographic questionnaire; during the second wave, they completed all methods for cognitive style and BFI-2 (in the mentioned order). The typical time of administration of the entire test battery was approximately 90 minutes for the first wave and 45 minutes for the second wave. All participants have given online informed consent.

Due the ongoing COVID-19 pandemic in the Czech Republic, the testing was online (with unified experimental conditions, such as computer peripheralsp). All methods were adapted for online testing in Hypothesis software allowing reliable capture of response times of all performance-based methods [117].

### Participants

Out of the pool of 600 participants, we collected data from 392 participants in total (380 in the first wave, 217 in the second wave). 12 participants participated only in the second wave, therefore the total number of participants is higher than a number in the first way. Eight additional participants were removed because of their high number of invalid answers (see Appendix 2 in S1 File); 384 participants were included in the analysis (see Table 1). Since not all participants answered all methods (some participants omitted them probably due to the online experimental settings, many participants were active only during first wave), only 116 participants passed all methods in both waves. However, each method was answered by at least 196 participants. And more importantly, methods for assessing cognitive style were fulfilled by 233–354 participants in the first wave of data collection which is crucial since these data were used for most of the following analyses. An a priori power analysis suggested that 250 participants should be sufficient for reliable estimation of crucial statistical procedures (see pre-registration for more details, https://osf.io/w483c). We therefore consider this sample size sufficient.

**Table 1:** Demographic characteristics of the participants.

**Age**	Range	18–90
	*Me* (*IQR*)	26 (13)
	*M* (*SD*)	30.35 (12.15)
**Gender**	Female	191 (49.74%)
	Male	101 (26.30%)
	*NA*	92 (23.96%)
**Education**	Elementary	12 (3.13%)
	High school	7 (1.83%)
	High school with graduation	120 (31.25%)
	University	153 (39.84%)
	*NA*	92 (23.96)
**Socio-economic status**	Poor	7 (1.82%)
	Lower mid	50 (13.02%)
	Mid	198 (51.6%)
	Upper mid	36 (9.38%)
	*NA*	93 (24.2%)
**Marital status**	Single	142 (36.98%)
	In relationship	76 (19.8%)
	married	65 (16.93%)
	Divorced	7 (1.82%)
	Widow	2 (0.52%)
**Number of siblings**	Range	0–8
	*Me* (*IQR*)	1 (1)

Note: *Me* = median, *IQR* = interquartile range, *M* = mean, *SD* = standard deviation, *NA* = missing values.

### Data analysis

Before the main analyses, the data cleaning was performed precisely as pre-registered (see Appendix 2 in S1 File). Since we removed a large number of participants in the ICAR number series subtest caused by technical issues in the SQL database (44% of participants had half and more missing values), we omitted it this method from further analysis.

The RTs from CFT1, CFT2, E-CSA-WA were modelled using Bayesian 4-parameter shifted Wald distribution process model with drift, alpha, theta and drift variability parameters [118]. The Gibbs sampling method with three Markov Chain Monte Carlo (MCMC) chains and 10,000 iterations was used. We set up the (rather non-informative) prior distributions based on quantitative pilot studies (for prior distributions, see https://osf.io/7ezax/).

We also used alternative procedures for modelling RT (ex-Gaussian distribution, lognormal response time item response theory models, Q-diffusion item response theory models and shifted Wald distribution process model), however, they did not fit the data well (see Appendix 3 in S1 File). The findings of alternative procedures are reported in the Appendix 4 in S1 File.

The CFT3 was modelled via the hierarchical Linear Ballistic Accumulator (LBA) [119] that models five parameters (drift rate for each accumulator, threshold, starting point, start point variability, non decision time) and priors for the group-level means for starting point, non decision time and treshold, and group level drift rates [120]. Hamiltonian Monte Carlo algorithm with 4 chains and 4,000 iterations (500 burn-in) was used. Having no information about the prior distribution beforehand, we used the prior distributions reported in Annis and colleagues [120].

ICAR subtests were estimated with Rasch models. The analysis of split-half reliability and the correlation analyses were bootstrapped with 10,000 iterations. Since the BFI-II had already been validated in Czech and yielded satisfactory internal consistency in our sample (extraversion ω = .872, agreeableness ω = .869, conscientiousness ω = .907, negative emotionality ω = .914, open mindedness ω = .881), we applied the subscale scores in the analyses calculated as arithmetic means.

The stability of constructs was verified via intraclass correlation coefficients (ICC) with two-way mixed effects and absolute agreement. The discriminant validity was verified via heterotrait-monotrait ratio of correlations (HTMT) and two-one-sided *t*-tests (TOST) [121]. In accordance with pre-registration, we specified for TOST the upper and lower equivalence bounds based on the smallest effect size of interest (SESOI) to -.25 and .25 as equivalents to practically null (absent) effect sizes. The concurrent and divergent validity was estimated within a multi-trait multi-method matrix (MTMM) and set of Spearman correlations. The predictive validity of the methods was verified with receiver operating characteristic (ROC) curves on the subsample of contast groups (participants with high vs. low socio-economic status).

In the structural equtation modelling (SEM), we used maximum likelihood estimation with robust (Huber-White) standard errors to handle missing values because the data showed multivariate non-normality [122] according to Henze-Zirkler’s test (*HZ* = 1.007, *p* < .001) as well as univariate non-normality of indicators according to Anderson–Darling tests (*p*s < .001), and because of the character of the response scale (seven categories) [123]. For evaluation of configural model fit, we applied the criteria proposed by Hu and Bentler [124]. In SEM, we used full information maximum likelihood (FIML) for dealing with missing values. In all other analyses, the pairwise approach was used. All analyses were performed in *R* (v4.1.1) [125] with packages *lavaan* [126], *semTools* [127], *TOSTER* [121], *psych* [128], *eRm* [129], and *irr* [130].

## Results

### Phase 0: Specific evidence of psychometric qualities

In the first step, the factor structure of AHS was verified with confirmatory factor analysis. Even though several alternative structures were proposed (including brief versions AHS-12 and AHS-4 [77], exploratory structural equtation modeling, and models obtained via exploratory and data-driven approach), none of them achieved the pre-registered criteria of model evaluation (*RMSEA* < .08, *SRMR* < .08, *CFI* > .90, *TLI* > .90; see Table 2). The internal consistency and average variance extracted (AVE) of all scales was also highly insufficient (locus of attention ω = .159, α = .315 *AVE* = .110; causal theory ω = .254, α = .314, *AVE* = .129; perception of change ω = .234, α = .238, *AVE* = .116; attitude toward contradictions ω = .550, α = .495, *AVE* = .249). Since the AHS demonstrated significant shortcomings in its factor structure and internal consistency and no model adjustment helped overcome these issues, we omitted AHS from further analysis (for results of AHS in all phases, see additional online supplementary materials at https://osf.io/7ezax/.

**Table 2:** Confirmatory factor analysis of AHS.

	*CFI*	*TLI*	*RMSEA* [*CI* 90%]	*SRMR*
mod1	.461	.395	.120 [.114, .127]	.116
mod2	.461	.385	.121 [.115, .128]	.116
mod3	.683	.605	.097 [.091, .104]	.083
mod4	.415	.359	.124 [.118, .130]	.117
mod5	.725	.622	.129 [.116, .143]	.110
mod6	.778	.704	.164 [.145, .183]	.092
mod7	.732	.632	.123 [.109, .137]	.089
mod8	.794	.382	.070 [.153, .252]	.032
mod9	.734	.695	.088 [.077, .098]	.115
mod10	.908	.880	.074 [.055, .093]	.086
mod11	.830	.747	.078 [.070, .086]	.051

Note: mod1 = original factor structure; mod2 = hierarchical factor structure (one second-order factor); mod3 = control for response bias (common method variance); mod4 = 1 factor structure; mod5 = original structure without items with low factor scores or high cross-loadings (data-driven); mod6 = 1 factor structure without items with low factor scores or high cross-loadings (data-driven); mod7 = AHS-12 version [77]; mod8 = AHS-4 version [77]; mod9 = 5factor model based on the exploratory factor analysis (all items); mod10 = 5factor model based on the exploratory factor analysis (3items per subscale); mod11 = Exploratory structural equtation modeling (ESEM-within-CFA) on original model (with target rotation); *RMSEA* = Root mean square error of approximation; *CI* = Confidence intervals; *CFI* = Comparative fit index; *TLI* = Tucker-Lewis index; *SRMR* = Standardized root mean square residual.

In the next step, we estimated empirical reliability from latent trait estimates and their corresponding standard errors (calculated from diffusion IRT model), i.e., empirical reliability of maximum a posteriori estimates, showing sufficient (reliability ≥ .70) for both subscales of CFT1 (local = .958, global = .983), CFT2 (local = .974, global = .954) and E-CSA-WA (analytic = .945, holistic = .942).

Split-half reliability was estimated only in ART. Split-half reliability was calculated on random halves using Guttman’s λ2. The reliability was identical to the pre-registered threshold for sufficient evidence of reliability (analytic = .50, holistic = .50).

### Phase 1: Stability of the construct

We found that ART indicated moderate test-retest reliability for the holistic subtest but insufficient reliability for the analytic subtest. CFT3 indicated good reliability. CFT1, CFT2 and E-CSA-WA indicated good reliability in median raw RTs and moderate reliability estimated according to the drift parameter from the Bayesian 4-parameter shifted Wald process model (except for the holistic subtest of E-CSA-WA, which, similarly to ART, showed ICC slightly below the pre-registered .50; see Tables 3 and 4).

**Table 3:** Intraclass correlation coefficients of ART and CFT3.

ART		CFT 3	
Analytic	Holistic	Analytic (hierarchical LBA drift)	Analytic (hierarchical LBA drift)
.444 [.259, .582]	.529 [.376, .645]	.791 [.702, .853]	.701 [.578, .788]

**Table 4:** Intraclass correlation coefficients of CFT1, CFT2 and E-CSA-WA.

	CFT 1		CFT 2		E-CSA-WA	
	Local	Global	Local	Global	Analytic	Holistic
Raw RT: Median	.900 [.865, .926]	.857 [.807, .894]	.871 [.701, .932]	.854 [.962, .919]	.795 [.608, .880]	.739 [.349, .868]
Bayesian 4-parameter shifted Wald: Drift	.505 [.329, .671]	.538 [.373, .660]	.570 [.351, .708]	.616 [.416, .740]	.573 [.374, .704]	.411 [-.039, .634]

### Phase 2: Discriminant validity with intelligence and personality

Concerning the discriminant validity of E-CSA-WA and CFT2 with personality traits, none of the HTMT values was above .90. Hence, all methods showed satisfactory discriminant validity (see Table 5).

**Table 5:** Discriminant validity of CFT2 and E-CSA-WA with personality traits.

Method	Indicator	extraversion	agreeableness	conscientiousness	negative emotionality	open-mindedness
CFT2: Local	Raw RT: Me	.101	.137	.060	.119	.134
	Bayesian 4-parameter shifted Wald: Drift	.237	.177	.172	.191	.294
CFT2: Global	Raw RT: Me	.102	.126	.060	.095	.136
	Bayesian 4-parameter shifted Wald: Drift	.191	.159	.147	.183	.256
E-CSA-WA: Local	Raw RT: Me	.144	.100	.085	.090	.122
	Bayesian 4-parameter shifted Wald: Drift	.106	.128	.122	.099	.151
E-CSA-WA: Global	Raw RT: Me	.121	.086	.087	.070	.118
	Bayesian 4-parameter shifted Wald: Drift	.163	.120	.074	.112	.121

The discriminant validity of ART, CFT1 and CFT3 with personality traits was verified using the TOST. All correlations with personality were lower than pre-registered .20 (see Table 6). The only exception was the drift parameter from CFT3 holistic subtests which correlated with conscientiousness, but its correlation only neglictibly exceeded the pre-registered treshold (*r* = .205). We can therefore conclude that all methods indicated satisfactory discriminant validity with personality traits.

**Table 6:** Discriminant validity of ART, CFT1 and CFT3 with personality traits.

Method	Indicator	extraversion	agreeableness	conscientiousness	negative emotionality	open mindedness
ART: Analytic	|ΔM|	.106	.075	.192	-.059	.052
ART: Holistic	|ΔM|	.077	.079	.064	-.038	-.021
CFT1: Local	Raw RT: Me	-.033	.154	.101	-.055	-.022
	Bayesian 4-parameter shifted Wald: Drift	.117	-.074	-.108	.012	-.010
CFT1: Global	Raw RT: Me	-.056	.161	.106	-.048	-.050
	Bayesian 4-parameter shifted Wald: Drift	-.043	-.006	-.068	.023	-.006
CFT3: Analytic	Hierarchical LBA: Drift	-.137	-.086	-.080	.067	-.134
CFT3: Holistic	Hierarchical LBA: Drift	.056	-.090	.205 *	-.061	.063

Note

* = insignificant TOST results (i.e., practically significant association).

The discriminant validity of all methods with intelligence was verified using TOST. CFT1 indicated weak negative correlations with matrix reasoning for both raw RTs and drift parameters (quicker participants in both subscales were more successful in matrix reasoning). These associations, however, were contained within the equivalence range, and therefore practical significance was not established. Associations between CFT2, CFT3 and E-CSA-WE were generally much lower and therefore also practically insignificant for both RTs and drift parameters (see Table 7). Nevertheless, CFT2 indicated practically significant and potentially problematic associations with the rotation subtest according to the alternative RT estimations (i.e., theta parameters of diffusion IRT models and lognormal RT IRT models; see Appendix 4, Table S3D in S1 File). Finally, the holistic subtest of ART indicated statistically and practically significant negative association with the rotation subset of ICAR. Participants who were more accurate in drawing relative lines in ART were also more successful in rotation. Since the discriminant criterion with intelligence is crucial, and ART also revealed some issues with stability, we omitted this method from the next phase of the validation process (for results of ART in all phases, see additional online supplementary materials at https://osf.io/7ezax/.

**Table 7:** Discriminant validity with intelligence.

Method	Indicator	Matrix	Rotation
ART: Analytic	|ΔM|	-.228	-.248
ART: Holistic	|ΔM|	-.247	-.360 *
CFT1: Local	Raw RT: Me	-.248	-.175
	Bayesian 4-parameter shifted Wald: Drift	.256	.161
CFT1: Global	Raw RT: Me	-.266	-.168
	Bayesian 4-parameter shifted Wald: Drift	.204	.128
CFT2: Local	Raw RT: Me	.054	.115
	Bayesian 4-parameter shifted Wald: Drift	.029	.047
CFT2: Global	Raw RT: Me	.130	.157
	Bayesian 4-parameter shifted Wald: Drift	-.042	-.064
CFT3: Analytic	Hierarchical LBA: Drift	-.116	-.081
CFT3: Holistic	Hierarchical LBA: Drift	-.048	-.025
E-CSA-WA: Local	Raw RT: Me	.075	.086
	Bayesian 4-parameter shifted Wald: Drift	.057	.105
E-CSA-WA: Global	Raw RT: Me	-.006	-.049
	Bayesian 4-parameter shifted Wald: Drift	-.053	-.024

Note

* = insignificant TOST results (i.e., practically significant association).

### Phase 3: Concurrent and divergent validity

The results of MTMM suggest that remaining four methods measure entirely different traits (i.e., the associations between related analytic/holistic subtests from various methods are not satisfactory). Similarly, low associations were found also for the same method-different trait values, (i.e., the association between analytic and holistic subtest within a single measure), and for different method-different trait values (i.e., associations between analytic/holistic subtest from different methods which should not be related; see Table 8).

**Table 8:** Multi-trait multi-method matrix for CFT1, CFT2, CFT3 and E-CSA-WA.

Indicator of RTs	Same Trait-Different Method	Same Method-Different Trait	Different Method-Different Trait
Bayesian 4-parameter shifted Wald: Drift	.087	.276	.112

To analyse specific associations between methods in more detail, we performed additional correlation analyses (see Table 9). It is evident that CFT3 was not associated with any other measure since all its correlations were lower than .12. Although the associations between CFT1, CFT2 and E-CSA-WA at the RT level were higher than the pre-registered threshold of .30, these results were not replicated for drift parameters. Lack of association between the drift parameters of various methods indicates that CFT3 and CFT1 represent different aspects of AH. Even though CFT2 and E-CSA-WA showed weak associations with drift parameters, they cannot be interpreted in terms of satisfactory concurrent validity, and therefore, most likely also measure different facets of AH.

**Table 9:** Spearman’s rank correlation coefficients between related AH subtests.

Method	Indicator	CFT2	CFT3	E-CSA-WA
CFT1: Local	Raw RT: Me	.38 [.24, .48] ***	-	.31 [.15, .39] ***
	Bayesian 4-parameter shifted Wald: Drift	.09 [-.04, .24]	.06 [-.05, .18]	.18 [.05, .29] *
CFT1: Global	Raw RT: Me	.33 [.22, .45] ***	-	.41 [.30, .49] ***
	Bayesian 4-parameter shifted Wald: Drift	.08 [-.07, .23]	.03 [-.08, .17]	.06 [-.06, .18]
CFT2: Local	Raw RT: Me	-	-	.36 [.20, .47] ***
	Bayesian 4-parameter shifted Wald: Drift	-	-.02 [-.19, .14]	.26 [.13, .38] ***
CFT2: Global	Raw RT: Me	-	-	.32 [.14, .42] ***
	Bayesian 4-parameter shifted Wald: Drift	-	-.04 [-.16, .13]	.23 [.12, .35] ***
CFT3: Analytic	Hierarchical LBA: Drift	-	-	.12 [-.03, .20]
CFT3: Holistic	Hierarchical LBA: Drift	-	-	.10 [-.04, .22]

Note

*** = *p*-value < .001

** = *p*-value < .01

* = *p*-value < .05.

Even thought the MTMM revealed the very small values in the same method-different trait values, we also report the associations between analytic and holistic subtests within each method separately because they provide more evidence about dimensionality of the construct (see Table 10). CFT1, CFT2 and E-CSA-WA showed very strong associations between both subtests for RTs, and subtests of CFT2 and E-CSA-WA remained highly correlated, even for drift parameters. On the other hand, the CFT3 showed negative associations between subtests (this fact caused the low same method-different trait value since other subtests from other three methods were associated). This suggests that CFT1 might effectively distinguish between analytic and holistic dimensions, whereas the assumption of two dimensions might be violated for CFT2 and E-CSA-WA. As for CFT3, the negative associations means that participants who score more on analytic subtest, score lower on holistic subtest (and vica versa) which suggests uni-dimensional structure of AH.

**Table 10:** Spearman’s rank correlation coefficients between subtests within one measure.

Method	Indicator	Associations between subtests
CFT1	Raw RT: Me	.83 [.78, .87] ***
	Bayesian 4-parameter shifted Wald: Drift	.20 [.08, .33] **
CFT2	Raw RT: Me	.89 [.86, .92] ***
	Bayesian 4-parameter shifted Wald: Drift	.62 [.52, .68] ***
CFT3	Hierarchical LBA: Drift	-.38 [-.51, -.26] ***
E-CSA-WA	Raw RT: Me	.83 [.78, .86] ***
	Bayesian 4-parameter shifted Wald: Drift	.56 [.46, .66] ***

Note

*** = *p*-value < .001

** = *p*-value < .01

* = *p*-value < .05.

### Phase 4: Predictive validity

The final phase of the validation process attempted to verify the predictive validity of methods. According to some evidence, social class should affect AH similarly to other cultural influences [131]. Persons from lower social classes should be more holistic and less analytic than persons from higher social classes. For this purpose, we split socio-economic status into two extremes: poor and lower mid socio-economic status (*N* = 57) on one side and upper mid socio-economic status (*N* = 36) on the other. We conducted a ROC curve and calculated area under the curve (AUC) to assess the ability of the methods of interest to discriminate between high and low socio-economic status.

Only the CFT1 local subtest met the recommended criteria of the acceptable discrimination (i.e., AUC > .70) at the RT level in the expected direction. However, the remainder of AUC values for both raw RT level and drift parameter were much smaller and thus hardly interpretable, especially since some of the means of drift paramters and RTs were opposite to the expected results (see Table 11).

**Table 11:** Predictive validity of measures.

Method	Indicator	Lower mid *M*	Expected direction	Upper mid *M*	*AUC*	95% *CI*
CFT1: Local	Raw RT: Me	1.139	>	0.996	.722	.612, .831
	Bayesian 4-parameter shifted Wald: Drift	4.134	<	4.327	.626	.500, .753
CFT1: Global	Raw RT: Me	1.012	<	0.874	.326	.210, .442
	Bayesian 4-parameter shifted Wald: Drift	3.893	>	3.900	.489	.349, .628
CFT2: Local	Raw RT: Me	2.229	>	2.069	.622	.469, .776
	Bayesian 4-parameter shifted Wald: Drift	2.455	<	2.525	.552	.392, .713
CFT2: Global	Raw RT: Me	2.244	<	2.062	.367	.214, .521
	Bayesian 4-parameter shifted Wald: Drift	2.533	>	2.664	.432	.271, .594
CFT3: Analytic	Hierarchical LBA: Drift	0.812	<	0.774	.509	.367, .651
CFT3: Holistic	Hierarchical LBA: Drift	2.285	>	2.372	.455	.320, .590
E-CSA-WA: Local	Raw RT: Me	1.533	>	1.328	.625	.498, .751
	Bayesian 4-parameter shifted Wald: Drift	2.932	<	3.099	.576	.442, .711
E-CSA-WA: Global	Raw RT: Me	1.293	<	1.195	.435	.307, .563
	Bayesian 4-parameter shifted Wald: Drift	2.243	>	2.507	.377	.247, .507

Note: *M* = mean; *AUC* = Area Under the Curve; *CI* = Confidence Intervals.

## Discussion

### Psychometric properties of developed AH instruments

This article presented psychometric properties of six proposed methods for measuring AH. The FLT appeared to be a very promising successor of the Witkin’s rod-and-frame test. However, our computer-based adaptation demonstrated a problem with the stability of the construct in time and an undesirable correlation with general intelligence, namely with its spatial ability subtest. Since no previous study has provided sufficient evidence for the validity of this method, in the context of this study, we cannot recommend it for further use. It appears that the rod-and-frame principle might be an interesting indicator of spatial ability, but its informative value regarding analytic and holistic cognitive styles remains ambiguous.

The E-CSA-WA, however, indicated moderate test-retest reliability, absence of association with personality and intelligence, and very weak concurrent validity with CFT1 and CFT2. Together with previous evidence of validity and reliability [57, 59] and a sufficient number of items per subscale for reliable RT estimation, we may, with certain reservations, consider it a valid method and recommend it (and the principle of *embedded figures* behind the instrument) for further use in assessing an individual’s cognitive style. However, the method’s moderate correlation between both subtests might suggest a certain dependence between analytic and holistic modes of processing information, which represents a certain limitation which should be further studied.

Regarding *Navon figures*, three modifications of this test were used (CFT1, CFT2 and CFT3). All relevant psychometric properties of CFT1 were found to be satisfactory. Even though this instrument indicated weak association with intelligence, it was not practically significant. Both subtests were only weakly associated with each other and hence we can recommend CFT1 for further use. However, since CFT1 contains a small number of items (although for traditional RT analysis, it might be considered satisfactory [105, 132]), estimation of the drift parameters in the shifted Wald model might be unreliable, and adding more items per subtest is desirable. To reflect this in future research, at least fifty items per subtest are recommended for a reliable estimation of drift parameters within shifted Wald distribution [133].

CFT2 also satisfied almost all validity and reliability criteria (with the exception of high correlation in its subtests; similarly to E-CSA-WA). CFT2, however, yielded some inconsistencies in the results which required investigation before the use of this instrument as an indicator of AH. The accuracy of CFT2 indicated higher variability in responses and was generally lower than in other instruments. In relation to this, the alternative RT estimations which take into account difficulty, for example, lognormal response time item response theory models and Q-diffusion item response theory models, also indicated a slight improvement in model fit (and therefore more reliable estimation). Even though we still cannot consider these estimations reliable, they indicate that the CFT2 has a practically significant association with intelligence (rotation subtest of ICAR, see Table S3D in Appendix 4 in S1 File). Is it therefore possible that when the difficulty of the tasks increases, solving them automatically overlaps with cognitive ability? If this is true, AH measurement must rely on simplier tasks to keep its discriminant validity with cognitive ability such as intelligence. We believe that the principle behind CFT2 needs further examination to verify whether the incorporation of more challenging tasks generates associations with general intelligence.

The CFT3, overall possessed good psychometric properties. Its stability in time was very high, probably because it is based on a different principle than the previous two Navon hierarchical figures (i.e., similarity matching task). It was not associated with intelligence and most of personality traits. Even thought it was slightly associated with conscientiousness (more holistic people were more conscientiousness), the association was very low and did not yeopardize its discriminat validity. Furthermore, with respect to the observed systematic cultural differences in the Big Five personality traits [134], our finding appears to be logical. After a few modifications, even this instrument can be considered for further use. The crucial modification should lie in the increase of number of tasks, since LBA generally needs more items to be reliable [119].

Our findings indicate the failure of self-report *AHS* to pass the first criterion of the valid factor structure. Since repeated verification of factor structure on different samples from multiple cultural groups is considered necessary when establishing the validity of questionnaires which measure cross-cultural constructs [86], its validity might be compromised. Of course, it is possible that our findings are specific for Czech samples, but since the prior evidence of factor structure and cross-cultural measurement invariance is scarce in the literature, we cannot recommend using of AHS self-report questionnaire in the research in its current form. Future validation research on various cultural samples is needed.

### Common shortcomings of AH instruments and future research

Although in the previous chapter we recommended three methods and the principles behind them for reliable AH measurement in the future (CFT1, CFT3, E-CSA-WA) and one method for deeper inspection of the link between difficulty in AH tasks and general intelligence (CFT2), we also identified some of their key limitations. These issues do not necessarily jeopardize the validity or reliability of the methods, because they may simply stem from the insufficiently substantiated theoretical background of AH research. These issues also might represent the future research in the AH field.

The first limitation relates to the stability of the AH construct. CFT1, CFT2 and E-CSA-WA only tightly exceeded the pre-registered threshold of ICC > .50 for drift parameters estimated within Bayesian 4-parameter shifted Wald process models (the holistic subtest of the E-CSA-WA was slightly below this threshold). Good stability was shown only in the CFT3 that was not based on the Navon search task but on the Navon similarity matching task. These results were not surprising, as a considerable number of questions have been recently raised about the stability of the AH concept. For example, Zhang [32] argued that cognitive styles are inherently a dynamic phenomenon, and Kozhevnikov and colleagues [4] emphasized a more task-dependent character in cognitive styles. Many situational factors are also considered to potentially affect the scores in perceptual tasks. For instance, RT for *Navon figures* may be affected by the participant’s current mood (positive moods are more likely to elicit a global level of processing, whereas negative moods lead to a local level of processing [e.g., 135, 136]. Our original results supported the theoretical models, suggesting a dynamic change in the AH level rather than traditional views on a cognitive style as an entirely stable trait. Further research manipulating with the length of test-retest measurement and examining the factors influencing the change in the level of AH (such as the effects of training or the emotional state) can enrich the current knowledge about the stability of analytic and holistic cognition.

The second limitation relates to the dimensionality of AH. The correlation analysis revealed that the methods are not effective in distinguishing between their analytic and holistic subtests (apart from CFT3). It is possible that E-CSA-WA and CFT2 both measure one-dimensional constructs with two slightly different tasks (or that both dimensions underline a single second-order factor). What seems probable is that analytic and holistic styles do not represent orthogonal dimensions but are at least to some extent associated with each other. Other explanation might lie in “meta-style” called flexibility-rigidity [137]. It is possible that this style highly saturates the scores in E-CSA-WA and CFT2 and, consequently, many participants with higly flexibility style can obtain high scores in both subtests, whereas the other with high rigity have low levels in both subtests. It is a question whether this finding should be considered a limitation or an immanent feature of AH measure on individual level [cf. 82, 138]. Further research can attempt to distinguish analytic and holistic subtests more satisfactorily from each other. This distinction could be pursued with eye-tracking research measuring dwell time spent on background and dominant objects. From the findings, analytic components might emphasize a focus on detail (simple figures which must be identified in complex figures should differ only in small details) and holistic parts might incorporate even more complex and embedded backgrounds for figures. However, from the current theoretical approaches in combination with our empirical findings, it is impossible to decide whether analytic and holistic subtests should be (positively) correlated, as this association was beyond the scope of previous research and must be replicated (besides as a consequence of the usage of derived indices).

The third limitation is in divergent validity. The MTMM showed that the methods do not effectively distinguish between subtests. Concerning concurrent validity, deeper inspection using correlation analyses revealed that CFT1 and CFT2 were associated only weakly (*r*s < .30) with E-CSA-WA and that CFT2 and CFT1 did not correlate at all. CFT3 also did not relate to any other methods. These results agree with previous research which revealed only weak associations between various AH instruments and between modified versions of an instrument [e.g., 50, 64, 66, 70, 72, 82, 83]. These findings are strongly against a two-dimensional AH theoretical model, which we suggest should be potentially revised with respect to our present findings. This is also in line with some other research which already found a two-dimensional model of AH unsuitable and simplistic [e.g., 9, 82, 103, 139]. Our results can also explain why some studies showed contradictory or ambiguous results in the flagship of AH research–East-West cross-cultural comparisons [e.g., 9, 102, 103, 132, 140–142].

Future research can be inspired by for instance complex multilevel hierarchical models which were proposed to deal with a multitude of cognitive style models but not specifically to the AH dimension. For example, Kozhevnikov and colleagues [4] described four main clusters of cognitive style, namely context dependence/independence, rule-based vs. intuitive processing, integration vs. compartmentalization, and internal vs. external locus of processing, which can manifest at four hierarchically sorted levels: perception, concept formation, higher-order cognitive processing and metacognitive processing. It is thus possible, that AH methods measure to some extent independent facets of the AH construct or even entirely independent constructs which manifest similarly in cross-cultural comparisons but are not related at the individual level.

Finally, the fourth limitation is in the lack of predictive validity. None of the methods were capable of detecting the differences between participants of low and high socioeconomic status, and some of the statistically significant differences even indicated opposite directions (i.e., participants with higher socio-economic status showed higher levels of both holistic and analytic cognitive style). One group being higher in both subtests than the other is one of the possible outcomes of group comparisons and does not necessarily mean that instruments measure ability rather than style or trait. For example, Lee and colleagues [143] compared holistic and analytic thinkers (based on *categorization/triad task*) and found that holistic thinkers were quicker in both local and global subtests of hierarchical figures. The true reason behind these findings, however, is most likely the uncertain dimensionality of the construct and should be considered a topic for future research.

### Study limitations

The presented study has several limitations. First, we were not able to obtain the pre-registered number of participants (*N* = 500). Even though a priori power analysis suggested that 250 observations should be sufficient for the planned techniques of reaction times modeling, especially for analyses based on structural equation modeling, the final sample size was relatively small and could decrease the statistical power. Second, we observed relatively high number of missing values in the dataset (most participants omitted some methods) which was most likely caused by online administration. We also had to removed ICAR number series subtest as a result of a technical issues during data collection. Third, since the AH is mainly cross-cultural theory, its predictive validity lies in cross-cultural comparison and not comparisons within a single cultural group. Usage the socioeconomic status as a main criterion of predictive validity is another limitation of this study. Hence, we must conclude that the predictive validity of the proposed instruments remains unknown and further robust cross-cultural validation of the instruments is desirable.

## Supporting information

S1 File (DOCX)
