# Supplementary material for: Analytic and holistic cognitive style as a set of independent manifests: Evidence from a validation study of six measurement instruments
Source: PLoS One. 2023 Jun 13;18(6):e0287057. doi: 10.1371/journal.pone.0287057 (PMC10263325; doi:10.1371/journal.pone.0287057)
Supplement: S1 File — (DOCX) [file pone.0287057.s001.docx]

# Appendix 1: Deviations from Pre-Registration

**Table S1A:** Deviations from pre-registration

| Category | Pre-registered | Conducted | Justification |
| --- | --- | --- | --- |
| Sample size | 500 participants. | 392 participants. | Although we were able to generate a pool of 600 participants exactly as pre-registered and motivated the participants with high financial reward, we were not able to gather data from all 500 participants, even after several reminders. However, 392 participants are considered still satisfactory in terms of statistical power and reliable estimation of parameters. |
| Sample characteristics | Partially represents Czech population. | Not representative. | Even though our sample contained hard to achieve cohorts and differed from traditionally used student populations, it cannot be considered even partially representative. |
| Methods | 24 items of ICAR 3D rotation. | Only 13 items of the 3D rotation subtest were used. | As a result of a technical issue, one specific answer was not saved. This concerned items 1, 6, 8, 10, 13, 15, 20 and 23. These were removed from analysis. |
| Methods | ICAR number series subtest. | Omitted from analysis. | As a result of a technical issue, many participants were not able to answer some number series. Because 44.16% of participants reported more than 50% of missing values, we removed this subtest from analysis. |
| Data cleaning | Removal of participants based on their person fits in CFT1, CFT2, E-CSA-WA. | Person fit statistics implemented in the LNIRT package, and manual elimination based on computationally problematic cases in estimating the diffusion IRT model. | We encountered several computational problems during estimation of the Q-diffusion IRT model. Respondents were extracted according to the error line which specifies a misfit case during estimation. When no indices of the misfit case were given and the model was not estimated, we applied person fit statistics from the LNIRT package and extracted participants on these indices. Then, we proceeded with estimation of the Q-model. |
| Data cleaning | Removal of multivariate outliers using Mahalanobis distance. | Not conducted. | This procedure was specified only for the AH scale. Since the removal of multivariate outliers did not change the results of a very poor fit, we did not include it in the article. |
| Data cleaning | Removal of univariate outliers using the ±3*IQR rule. | Not conducted. | This procedure was not suitable for most analyses. Because we performed a detailed pre-registered data cleaning procedure, we removed any other additional participants on the basis of this arbitrary rule. |
| Data cleaning | Removing RTs higher than 5s | Removed RTs higher than 10s in the case of CFT3. | The pre-registered criterion was related only to CFT1, CFT2 and E-CSA-WA and not to CFT3. Since we used LBA which requires RTs, we specified additional criteria purely for CFT3. We set this criterion to be higher (10s), because the instruction did not emphasise the reaction speed and the answering was based on higher cognitive processing than in previous methods. |
| RTs estimations | Ex-gaussian distributions, hierarchical IRT models and diffusion IRT models. | Application of shifted Wald and Bayesian shifted Wald models. | Because our methods indicated a lack of variability in accuracy, we also applied additional RT estimations which we did not know of during the process of pre-registration. These new models fit the data well, and we therefore reported them in the main article. |
| Data analysis | Thurstonian IRT model for the CFT3. | Hierarchical linear ballistic accumulator (LBA) | In questionnaires, Thurstonian IRT (TIRT) is applied in a forced-choice format. Because simulation and empirical studies imply that at least five traits should be included to address the ipsativity of the score, we could not use this approach. However, the LBA was created to handle such situations and therefore we applied it. |
| Data analysis | Not specified. | Bootstrapping with 10,000 iterations. | To obtain more accurate results and their confidence intervals, bootstrapping where possible (i.e., split-half reliability and correlation analyses) was performed. |
| Data analysis | Not specified | MLR estimator | Because the data showed multivariate non-normality, and response scales had seven categories, we applied an MLR in structural equation modelling. |
| Results | Removal of methods with ICC < .50 from further validation phases due its low stability in time. | ART and E-CSA-WA continued to the next validation phases. | We kept these methods in additional analyses since their stability was always only slightly below .50 for one subtest (ART analytic subtest ICC = .444, E-CSA-WA analytic subtest = .433). |
|  |  |  |  |
| Results | Verification of predictive validity through differences in social class (derived from the participant’s education, participant’s socioeconomic status and education level of parents. | Verification of predictive validity through differences in socioeconomic status. | We did not collect data on the educational level of the participants’ parents since this question was not specified during approval by the ethical committee. Socioeconomic status by itself should be a significant predictor of AH. |
| Results | Verification of differences in social classes using ANOVA and comparison of low social class and high social class through post-hoc tests. | ROC curves on two extremely contrasting groups (poor and lower mid socioeconomic status vs. upper mid socioeconomic status). | ANOVA was not used because we wanted to simplify the results. Based on the reviewer’s suggestion, we decided to use ROC curves that are more proper for verification of predictive validity in two contras groups (instead of one-tailed *t*-tests that can be found in online additional materials). |

# Appendix 2: Data cleaning

Data cleaning was performed precisely as pre-registered. We adopted the following arbitrary criteria as an assessment of invalid responses: RTs ms < 200 ms (= answer before stimuli appearance) or RTs > 5,000 ms (= absurdly high RT) in CFT1, CFT2 and E-CSA-WA (solely in the case of CFT3, the criterium was set to RTs > 10,000s since instruction did not emphasise the reaction speed and the answering was based on higher cognitive processing) and deviation > 150 px (= absurdly high |∆*M*|) in ART. In all methods, the participants with missing values (i.e., invalid answers or unfinished tests) greater than 50% were removed from further analysis. No other criteria for participant or response removal (e.g., analysis of outliers) were applied since the mechanical removal of values is not recommended for analysis of RTs (Ratcliff, 1993).

Some additional criteria were applied only for diffIRT and LNIRT estimation. In the case of diffIRT, participants were removed according to their estimates which were intractable. In the case of LNIRT, participants were removed according to person-fit statistics which revealed who had a posterior probability higher than 95% of aberrant response times or accuracy. The results of data cleaning are in Table 3:

**Table:** Removed answers and participants.

| **Measure** | **RT < 200 ms** | **RT > 5,000 ms** | **\|∆*M*\| > 150 px** | ***NA* > 50%** | **diffIRT** | **LNIRT** | **Total *NA*** |
| --- | --- | --- | --- | --- | --- | --- | --- |
| CFT1 W1 | 46 (0.51%) | 24 (0.26%) | - | 0 | 8 | 11 | 29.43% |
| CFT1 W2 | 8 (0.13%) | 8 (0.13%) | - | 1 (0.51%) | 1 | 2 | 49.74% |
| CFT2 W1 | 4 (0.02%) | 586 (2.84%) | - | 9 (3.49%) | 7 | 4 | 35.16% |
| CFT2 W2 | 0 | 333 (2.26%) | - | 6 (3.16%) | 0 | 1 | 52.08% |
| CFT3 W1 | 17 (0.30%) | 92 (1.92%) ** | - | 1 (0.35%) | - | - | 39.32% |
| CFT3 W2 | 3 (0.08%) | 38 (0.96%) ** | - | 0 | - | - | 54.17% |
| E-CSA-WA W1 | 95 (0.42%) | 848 (3.79%) | - | 27 (9.64%) | 2 | 8 | 34.11% |
| E-CSA-WA W2 | 45 (0.31%) | 307 (2.12%) | - | 6 (3.31%) | 0 | 2 | 54.43% |
| ART W1 | - | - | 149 (3.39%) | 12 (3.28%) | - | - | 7.81% |
| ART W2 | - | - | 44 (1.71%) | 3 (3.28%) | - | - | 45.05% |
| AHS W1 | - | - | - | 1 (0.29%) | - | - | 9.90% |
| AHS W2 | - | - | - | 0 | - | - | 45.57% |
| BFI-2 | - | - | - | 0 | - | - | 48.96% |
| ICAR matrices | - | - | - | 0 | - | - | 17.45% |
| ICAR rotation | - | - | - | 18 (5.68%) | - | - | 22.13% |
| ICAR numbers | - | - | - | 140 (44.16%) | - | - | 62.5% |

* note: RT = reaction time, ms = milliseconds, *|∆|* = absolute mean difference, *NA* = missing values, W1 = first wave of data collection, W2 = second wave of data collection, ** = the criterium was set up for 10,000s.

It is evident from Table 3 that a relatively small number of answers (*n*s ≤ 3.79%) and only a minority of participants (besides one exception *n*s ≤ 9.64%) were removed across all methods. One measure, however, indicate a higher rate of removed participants. We removed 44% of participants from the ICAR number series subtest as a result of a large number of participants being unable to send their answers for this subtest. Since such a huge number of removed participants could bias the result, we omitted the entire subtest from further analysis.

# Appendix 3: Evaluation of models fits

Each model of the five approaches was verified before main analysis. However, the only RT estimations which yielded satisfactory fit indices were the shifted Wald process model. This is not surprising since the diffusion IRT model and LNIRT were designed for methods which produce high variability in accuracy (Anders et al., 2016; Faulkenberry, 2017). Even so, the methods were rather easy for participants to answer (accuracy of CFT1 local: 96.8%, CFT1 global: 97.3%, CFT2 local: 86.9%, CFT2 global: 92.1%, E-CSA-WA analytic: 97.2%, E-CSA-WA holistic: 96.8%). Although ex-Gaussian distributions fit the data adequately, their fits were inferior compared to shifted Wald. Hence, the parameter estimates of the shifted Wald model may be considered the most reliable. Since the Bayesian 4-parameter shifted Wald model adds one extra parameter, its results should be more accurate (Steingroever et al., 2021). Its estimates also highly correlated with the estimates of the shifted Wald process model.

Ex-gaussian distribution was evaluated according to a posterior predictive check. A comparison of observed and simulated data did not demonstrate a strong model fit (but may be considered satisfactory, see Figures S2A-S2F). Regarding the hierarchical IRT model, each item of every method (with the exception of CFT2 local) indicated a non-normal residual distribution based on the results of a Kolmogorov-Smirnov test, suggesting poor model of diffusion IRT models was assessed by M_r_ statistics implemented in the diffIRT package. We encountered severe computational issues in estimating this statistic. Even though this procedure was computationally infeasible due to the higher number of items for CFT2 and E-CSA-WA, it was safe to assume that the model fits for the Q-diffusion IRT model were unacceptable. This finding was also supported by QQ plots, which demonstrated significant inconsistencies between the observed and predicted RTs for each item separately. To evaluate the shifted Wald model, three diagnostic tools proposed by Anders et al. (2016) were applied and *R* functions by Faulkenberry (2017) were adopted. First, the QQ plot of the estimated and predicted deciles of RT was examined for each item. No curvature was detected, indicating a good model fit. However, the model slightly underestimates the middling deciles of CFT 2 global and local and ECSA global (see figures S2G-S2L). Second, the residual distribution plot of each decile was examined. The residual distributions of each decile indicated positive skewness in the data since the magnitude of the residuals increased with the magnitude of RT (see Figures S2A-S2F). Third, the residual summary statistics for each participant were obtained. The average residuals yielded increased values, however, the correlation coefficient between the standard deviation of residuals and average residuals was low, indicating a good model fit. Unfortunately, the model fit for the Bayesian shifted Wald model has not yet been implemented in *R* packages. We assume that its model fits will be very similar to the shifted Wald model. A summary of the results of all approaches are given in Table S2A.

**Table S2A:** Evaluation of models fits

| **Model** | **Indicator** | **CFT1** | | **CFT2** | | **E-CSA-WA** | |
| --- | --- | --- | --- | --- | --- | --- | --- |
|  |  | Analytic | Holistic | Analytic | Holistic | Analytic | Holistic |
| LNIRT | Kolmogorov-Smirnov Test (% of items that have non-normally distributed residuals) | 100% | 100% | 90% | 100% | 100% | 100% |
|  | % of extreme residuals | 0% | 0% | 0% | 0% | 0% | 0% |
|  | % of misfitting items | 0% | 0% | 0% | 0% | 0% | 0% |
| diffIRT | M_r_ | 116237 | 29609 | computationally infeasible | | | |
|  | df | 116 | 116 | computationally infeasible | | | |
|  | *p*-value | < .001 | < .001 | computationally infeasible | | | |
|  | *AIC* | 836 | -270 | 26921 | 21499 | 9489 | 14989 |
|  | *BIC* | 1018 | -90 | 27349 | 21925 | 9920 | 15420 |
| Shifted-Wald distribution | ∆¯ | 1.27 | 1.18 | 0.86 | 0.86 | 0.78 | 0.75 |
|  | σ_x_ | 213 | 204 | 615 | 593 | 476 | 753 |
|  | ρ_∆σ_ | 0.08 | 0.04 | -0.13 | -0.16 | -0.13 | 0.13 |

**Figure S2A**: Model fit of CFT 1 Global using posterior predictive check

**
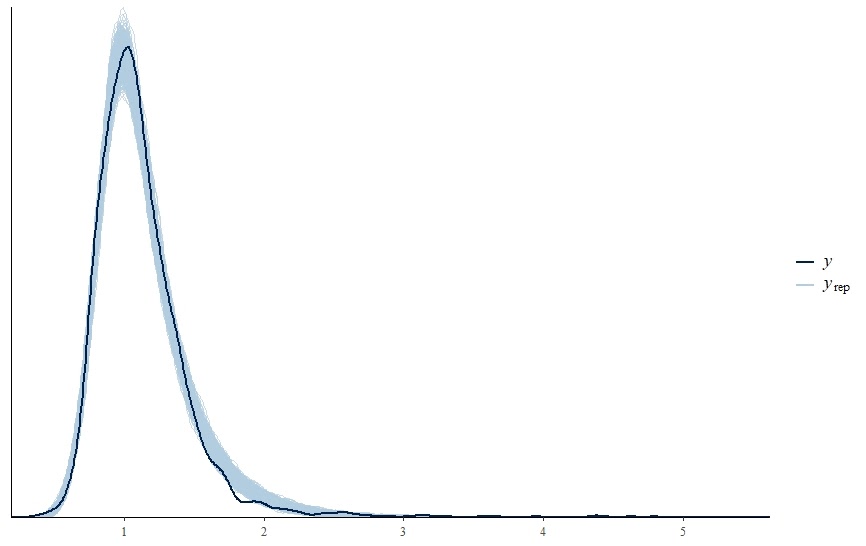
**

**Figure S2B**: Model fit of CFT 1 Local using posterior predictive check

**
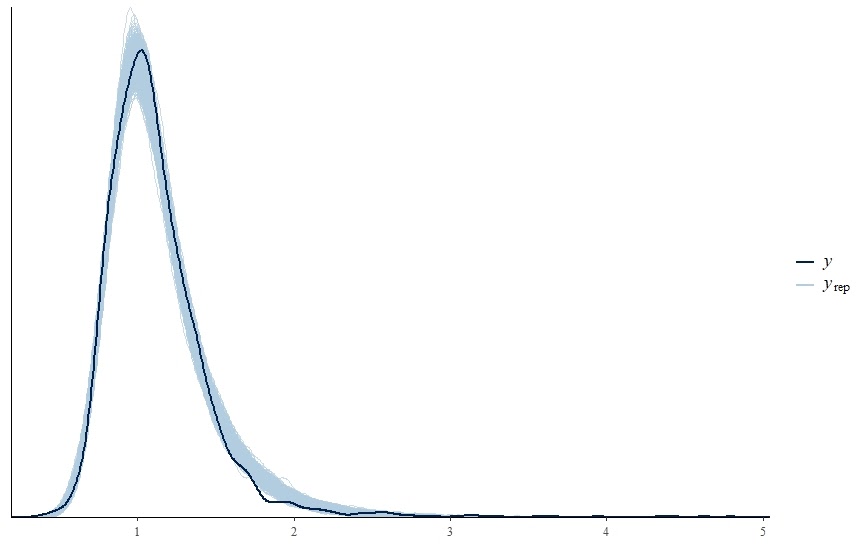
**

**Figure S2C**: Model fit of CFT 2 Global using posterior predictive check

**
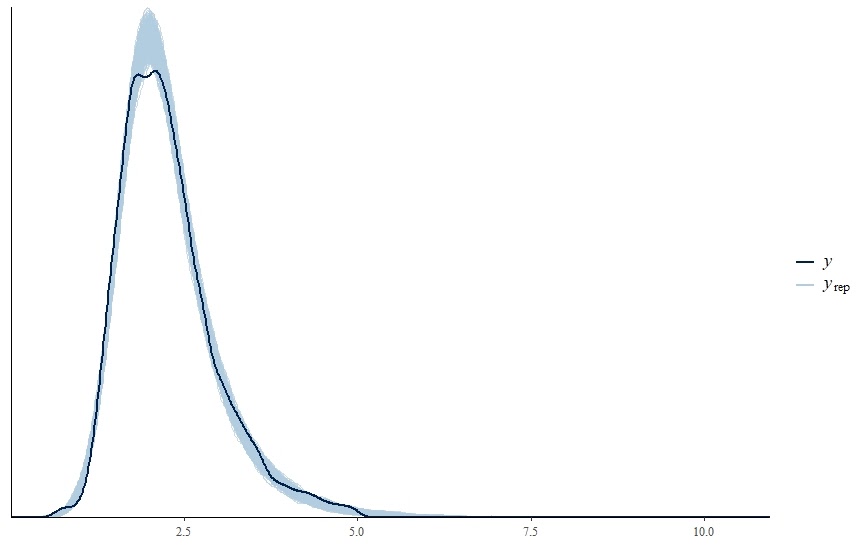
**

**Figure S2D**: Model fit of CFT 2 Local using posterior predictive check

**
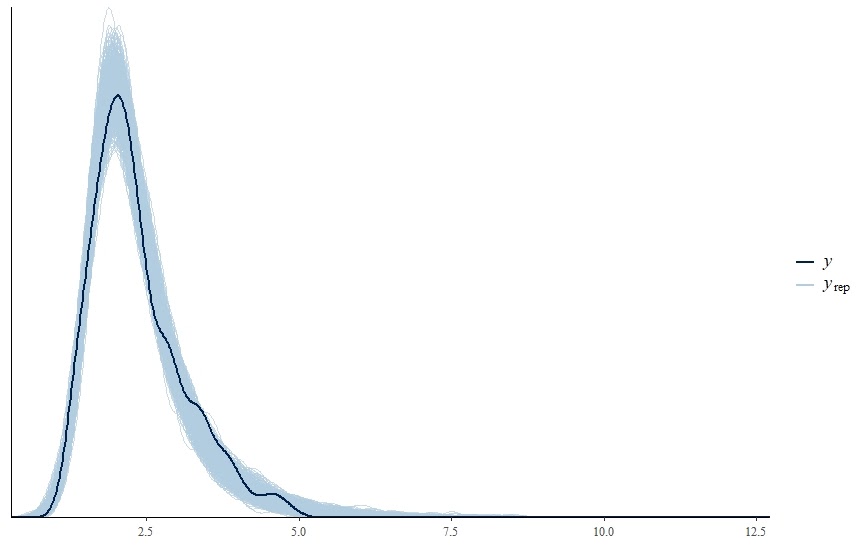
**

**Figure S2E**: Model fit of E-CSA-WA Holistic using posterior predictive check

**
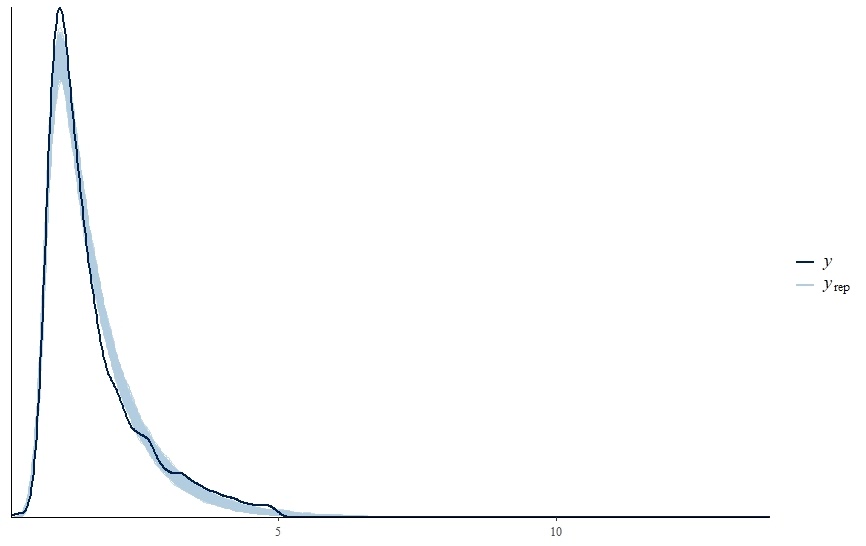
**

**Figure S2F**: Model fit of E-CSA-WA Analytic using posterior predictive check

**
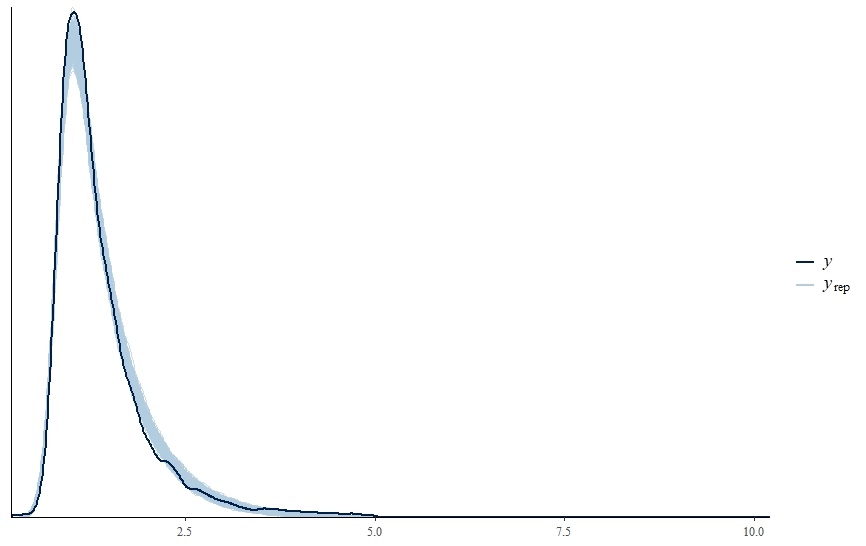
**

**Figure S2G**: Shifted-Wald model fit for CFT1 local


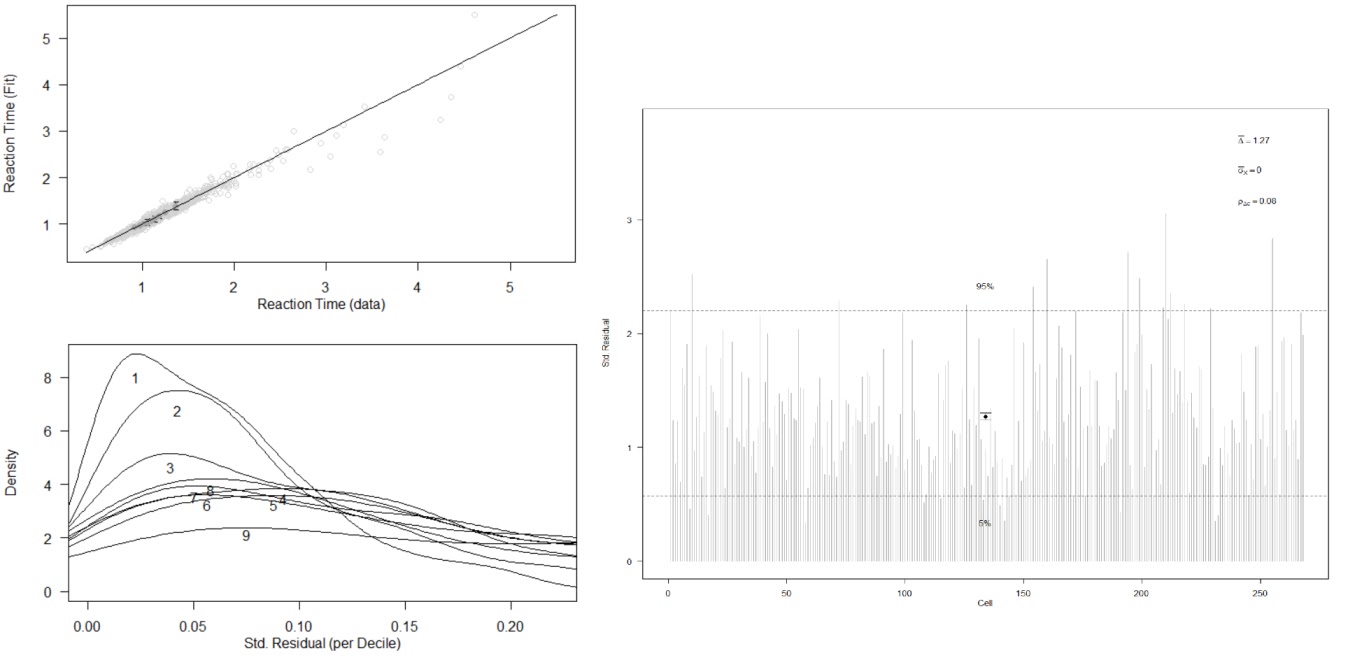


**Figure S2H**: Shifted-Wald model fit for CFT1 global


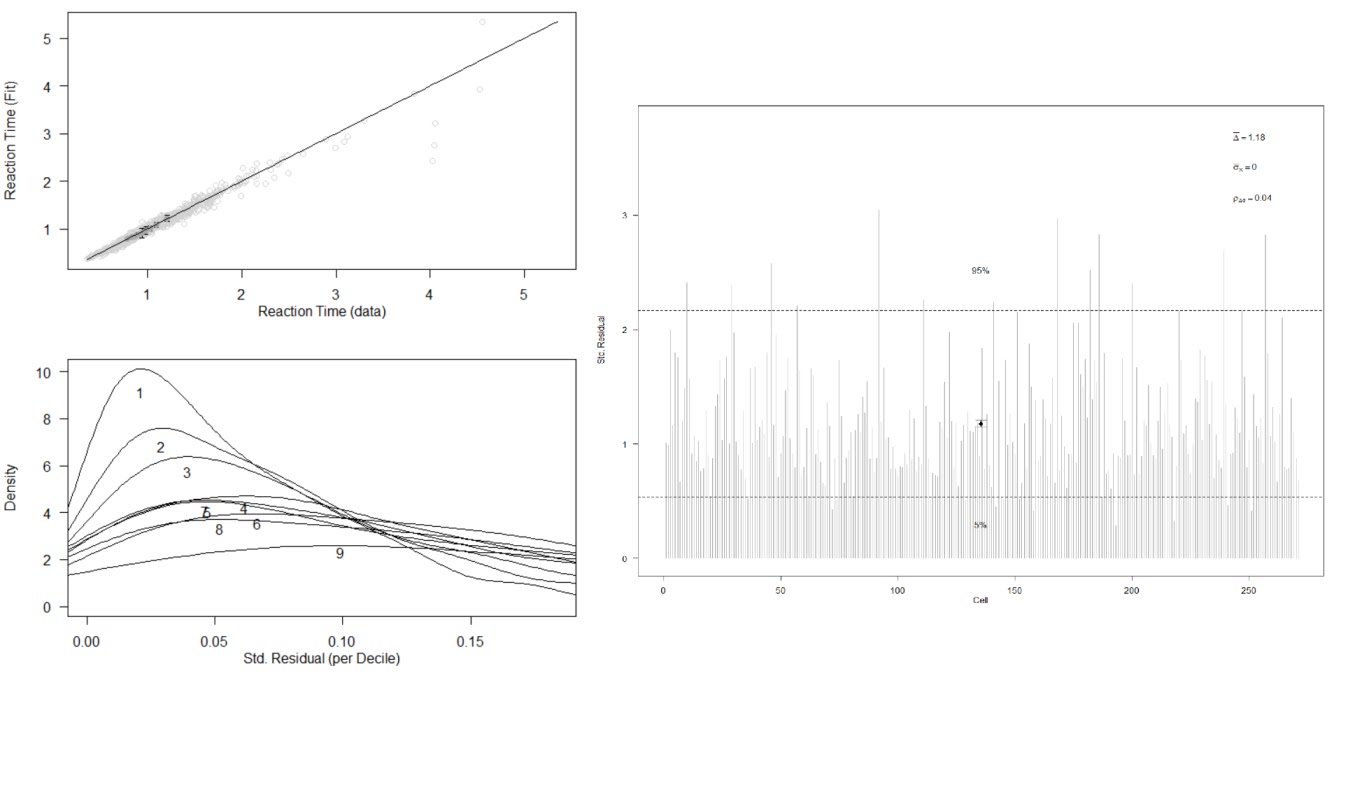


**Figure S2I**: Shifted-Wald model fit for CFT2 local


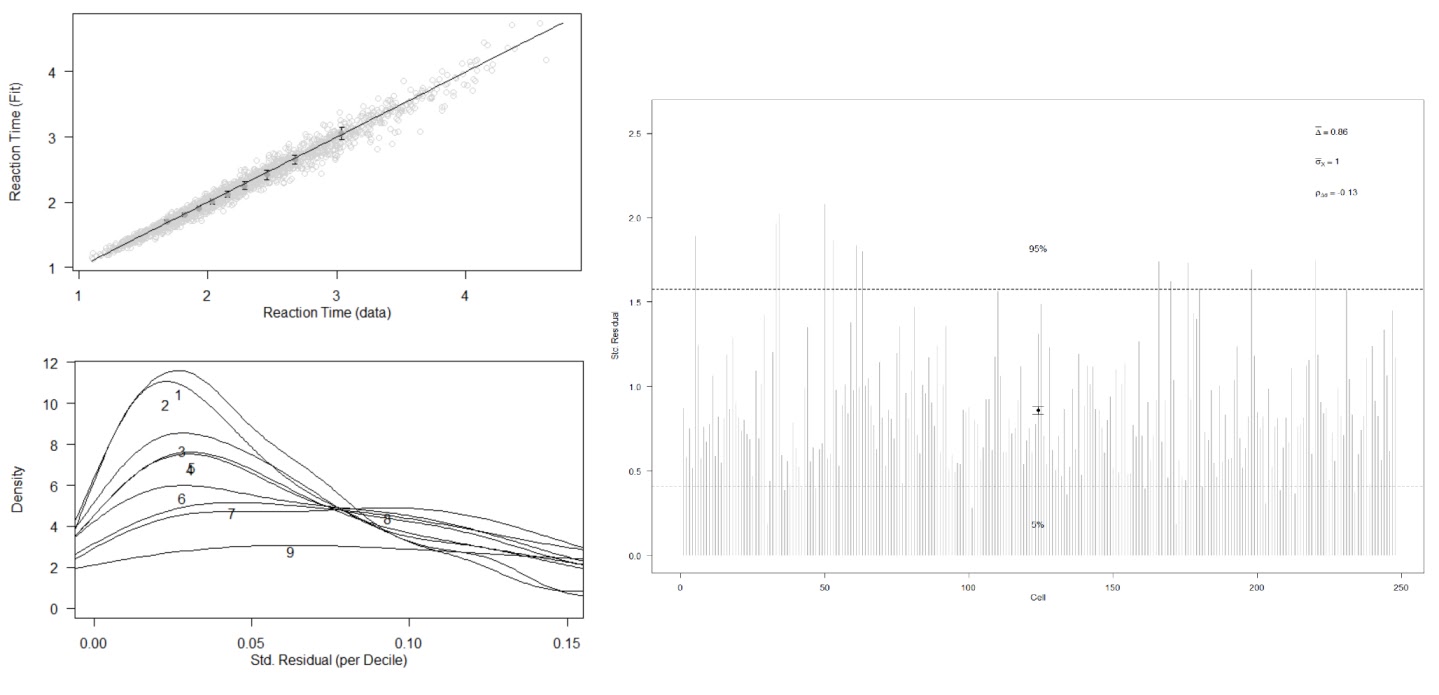


**Figure S2J**: Shifted-Wald model fit for CFT2 global


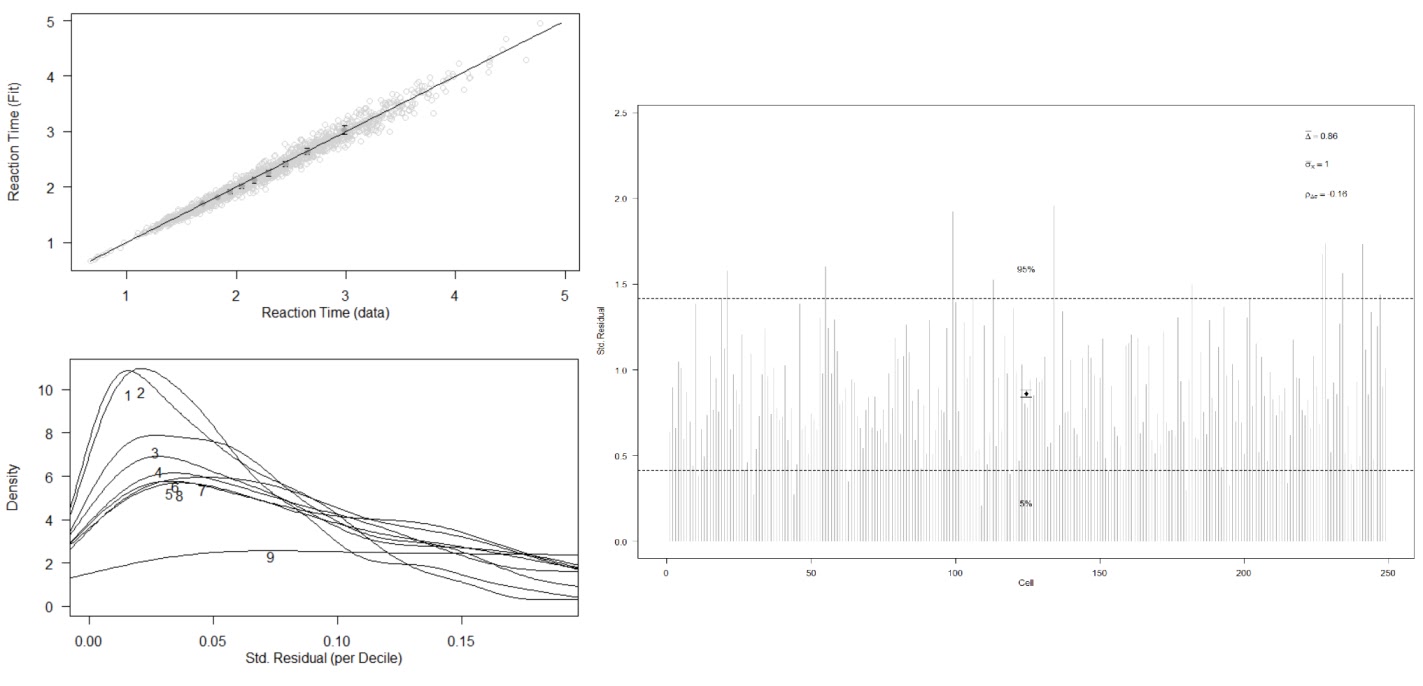


**Figure S2K**: Shifted-Wald model fit for E-CSA-WA local


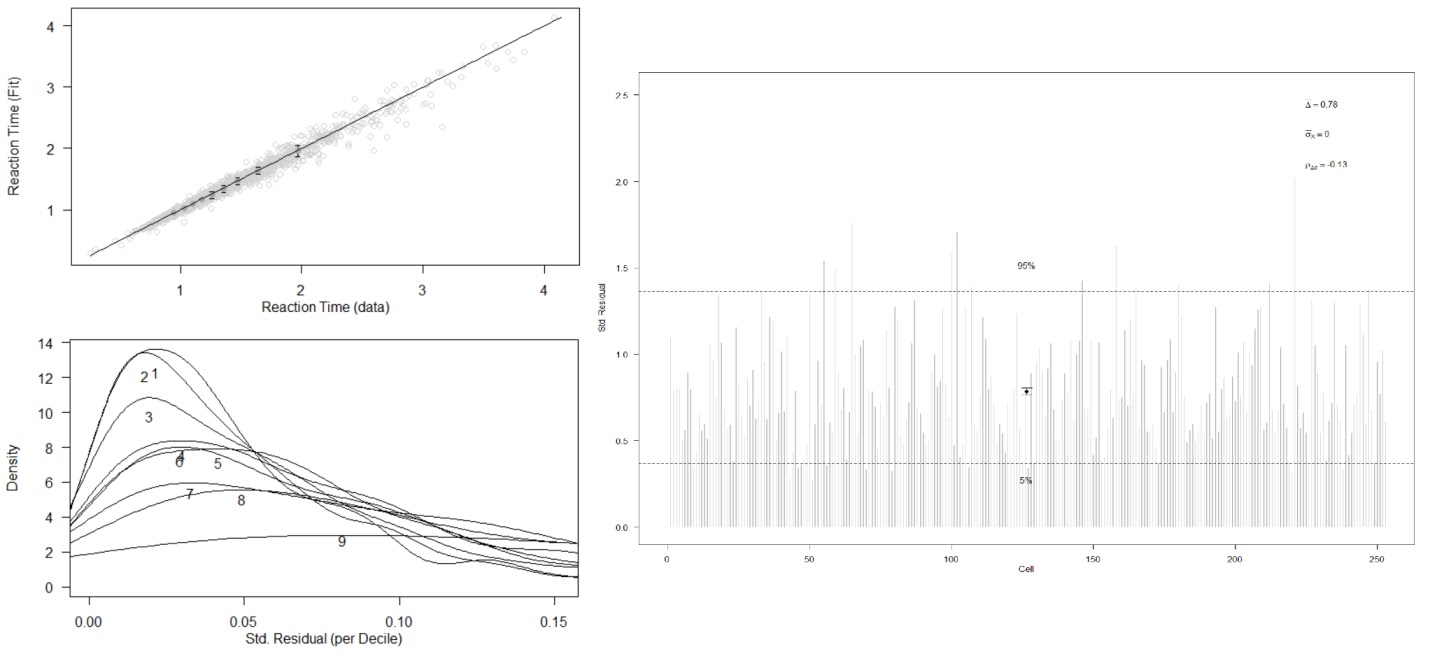


**Figure S2L**: Shifted-Wald model fit for E-CSA-WA global


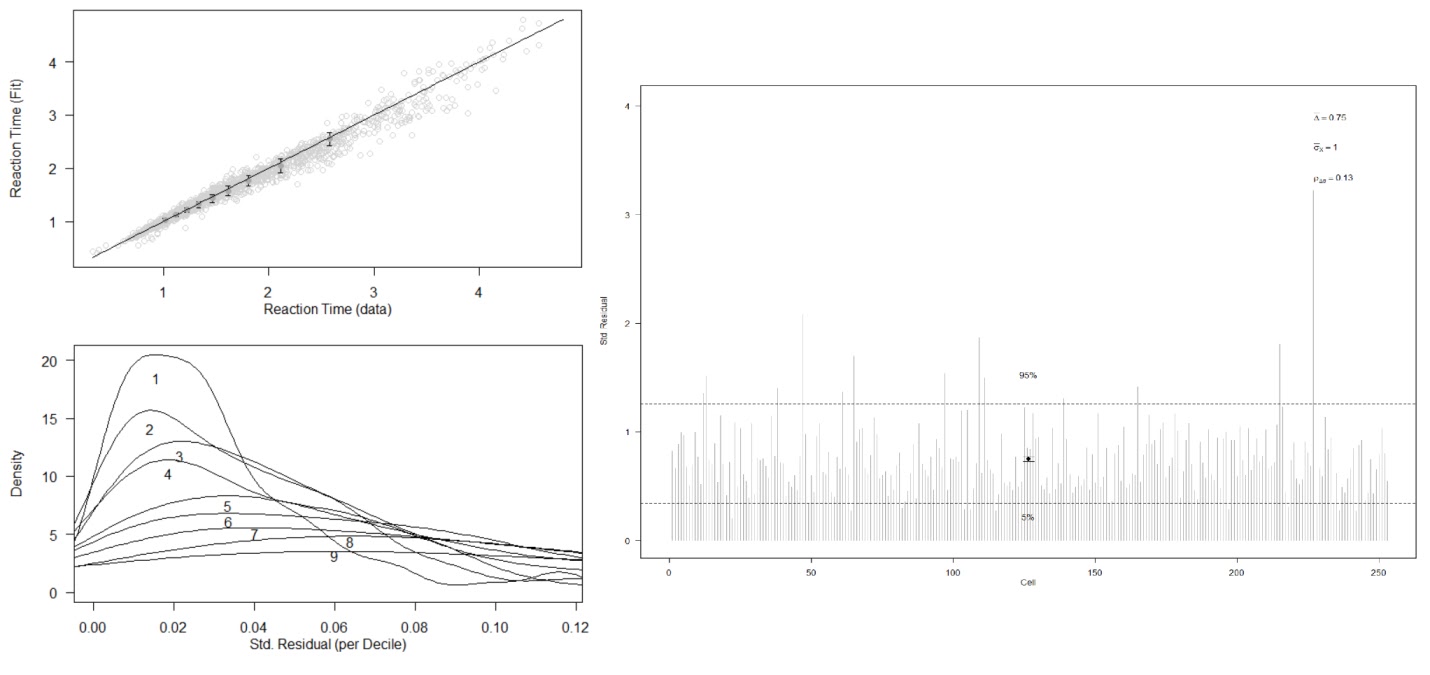


# Appendix 4: Robustness check

**Table S3A:** Intraclass correlation coefficients for CFT1, CFT2 and E-CSA-WA.

|  | CFT 1 | | CFT 2 | | E-CSA-WA | |
| --- | --- | --- | --- | --- | --- | --- |
|  | Local | Global | Local | Global | Analytic | Holistic |
| Raw RT: Median | .900  [.865, .926] | .857  [.807, .894] | .871  [.701, .932] | .854  [.962, .919] | .795  [.608, .880] | .739  [.349, .868] |
| Ex-Gaussian: Tau | .302   [.052, .487] | .255  [-.011, .451] | .469  [.265, .616] | .332  [.081, .516] | .451  [.053, .661] | .639  [.416, .765] |
| LNIRT: Theta | .062  [-.250, .298] | .057  [-.274, .302] | .740  [.639, .813] | .845  [.786, .888] | .474  [.268, .622] | .475  [.270, .622] |
| diffIRT: Theta | .168  [-.117, .381] | .282  [.030, .470] | .456  [.246, .608] | .690  [.572, .776] | .340  [.090, .523] | .514  [.328, .649] |
| Shifted-Wald: Drift | .215  [-.061, .420] | .492  [.310, .626] | .439  [.225, .593] | .389  [.158, .557] | .409  [.084, .607] | .463  [.250, .615] |
| Bayesian shifted-Wald: Drift | .505  [.329, .671] | .538  [.373, .660] | .570  [.351, .708] | .616  [.416, .740] | .573  [.374, .704] | .411  [-.039, .634] |

**Table S3B:** Heterotrait-monotrait ratio of correlations of CFT2 and E-CSA-WA with personality

| Method | Indicator | extraversion | agreeableness | conscientiousness | negative emotionality | open mindedness |
| --- | --- | --- | --- | --- | --- | --- |
| CFT2: Local | Raw RT: Me | .101 | .137 | .060 | .119 | .134 |
|  | Ex-Gaussian: tau | .556 | .390 | .553 | .389 | .428 |
|  | LNIRT: theta | .242 | .263 | .269 | .201 | .230 |
|  | diffIRT: theta | .084 | .156 | .113 | .092 | .110 |
|  | Shifted-Wald: Drift | .450 | .416 | .297 | .273 | .389 |
|  | Bayesian shifted-Wald: Drift | .237 | .177 | .172 | .191 | .294 |
| CFT2: Global | Raw RT: Me | .102 | .126 | .060 | .095 | .136 |
|  | Ex-Gaussian: tau | .398 | .271 | .261 | .242 | .347 |
|  | LNIRT: theta | .125 | .165 | .207 | .113 | .218 |
|  | diffIRT: theta | .080 | .091 | .090 | .062 | .075 |
|  | Shifted-Wald: Drift | .381 | .342 | .278 | .336 | .448 |
|  | Bayesian shifted-Wald: Drift | .191 | .159 | .147 | .183 | .256 |
| E-CSA-WA: Local | Raw RT: Me | .144 | .100 | .085 | .090 | .122 |
|  | Ex-Gaussian: tau | .196 | .183 | .172 | .105 | .138 |
|  | LNIRT: theta | .202 | .210 | .133 | .158 | .249 |
|  | diffIRT: theta | .166 | .178 | .127 | .128 | .193 |
|  | Shifted-Wald: Drift | .150 | .173 | .151 | .114 | .211 |
|  | Bayesian shifted-Wald: Drift | .106 | .129 | .114 | .101 | .147 |
| E-CSA-WA: Global | Raw RT: Me | .121 | .086 | .087 | .070 | .118 |
|  | Ex-Gaussian: tau | .234 | .200 | .144 | .189 | .150 |
|  | LNIRT: theta | .272 | .211 | .252 | .207 | .293 |
|  | diffIRT: theta | .250 | .204 | .134 | .141 | .171 |
|  | Shifted-Wald: Drift | .190 | .170 | .076 | .120 | .093 |
|  | Bayesian shifted-Wald: Drift | .163 | .116 | .076 | .108 | .118 |

**Table S3C:** Discriminant validity of ART, CFT1 and CFT3 with personality

| Method | Indicator | extraversion | agreeableness | conscientiousness | negative emotionality | open mindedness |
| --- | --- | --- | --- | --- | --- | --- |
| ART: Analytic | \|∆M\| | .106 | .075 | .192 | -.059 | .052 |
| ART: Holistic | \|∆M\| | .077 | .079 | .064 | -.038 | -.021 |
| CFT1: Local | Raw RT: Me | -.033 | .154 | .101 | -.055 | -.022 |
|  | Ex-Gaussian: tau | -.144 | .046 | .128 | -.067 | -.029 |
|  | LNIRT: theta | .111 | -.087 | .056 | .012 | .013 |
|  | diffIRT: theta | .132 | -.097 | .040 | -.041 | .019 |
|  | Shifted-Wald: Drift | .131 | -.012 | -.066 | -.010 | -.016 |
|  | Bayesian shifted-Wald: Drift | .115 | -.092 | -.104 | .021 | -.019 |
| CFT1: Global | Raw RT: Me | -.056 | .161 | .106 | -.048 | -.050 |
|  | Ex-Gaussian: tau | .081 | .027 | .128 | -.078 | .019 |
|  | LNIRT: theta | .050 | -.021 | -.095 | .027 | .105 |
|  | diffIRT: theta | .072 | .012 | -.039 | .009 | .111 |
|  | Shifted-Wald: Drift | -.008 | .026 | -.039 | .010 | .029 |
|  | Bayesian shifted-Wald: Drift | -.048 | -.026 | -.079 | .009 | -.002 |
| CFT3: Analytic | Hierarchical LBA: Drift | -.137 | -.086 | -.080 | .067 | -.134 |
| CFT3: Holistic | Hierarchical LBA: Drift | .056 | -.090 | .205 * | -.061 | .063 |

**Table S3D:** Discriminant validity with intelligence

| Method | Indicator | matrix | rotation |
| --- | --- | --- | --- |
| ART: Analytic | \|∆M\| | -.228 | -.248 |
| ART: Holistic | \|∆M\| | -.247 | -.360 * |
| CFT1: Local | Raw RT: Me | -.248 | -.175 |
|  | Ex-Gaussian: tau | -.124 | -.116 |
|  | LNIRT: theta | .067 | .101 |
|  | diffIRT: theta | .212 | .127 |
|  | Shifted-Wald: Drift | .145 | .100 |
|  | Bayesian shifted-Wald: Drift | .256 | .161 |
| CFT1: Global | Raw RT: Me | -.266 | -.168 |
|  | Ex-Gaussian: tau | -.180 | -.183 |
|  | LNIRT: theta | .121 | .164 |
|  | diffIRT: theta | .128 | .175 |
|  | Shifted-Wald: Drift | .164 | .098 |
|  | Bayesian shifted-Wald: Drift | .204 | .128 |
| CFT2: Local | Raw RT: Me | .054 | .115 |
|  | Ex-Gaussian: tau | .069 | .030 |
|  | LNIRT: theta | .264 | .362* |
|  | diffIRT: theta | .233 | .361* |
|  | Shifted-Wald: Drift | -.039 | .035 |
|  | Bayesian shifted-Wald: Drift | .029 | .047 |
| CFT2: Global | Raw RT: Me | .130 | .157 |
|  | Ex-Gaussian: tau | .036 | .100 |
|  | LNIRT: theta | .334 | .278 |
|  | diffIRT: theta | .253 | .248 |
|  | Shifted-Wald: Drift | -.048 | .120 |
|  | Bayesian shifted-Wald: Drift | -.042 | -.064 |
| CFT3: Analytic | Hierarchical LBA: Drift | -.116 | -.081 |
| CFT3: Holistic | Hierarchical LBA: Drift | -.048 | -.025 |
| E-CSA-WA: Local | Raw RT: Me | .075 | .086 |
|  | Ex-Gaussian: tau | .027 | -.016 |
|  | LNIRT: theta | .296 | .300 |
|  | diffIRT: theta | .181 | .230 |
|  | Shifted-Wald: Drift | .016 | .053 |
|  | Bayesian shifted-Wald: Drift | .057 | .105 |
| E-CSA-WA: Global | Raw RT: Me | -.006 | -.049 |
|  | Ex-Gaussian: tau | .058 | .020 |
|  | LNIRT: theta | .187 | .236 |
|  | diffIRT: theta | .109 | .099 |
|  | Shifted-Wald: Drift | -.033 | .022 |
|  | Bayesian shifted-Wald: Drift | -.053 | -.024 |

**Table S3E:** Multi Trait Multi Method Matrix for CFT1, CFT2, CFT3 and E-CSA-WA

| Indicator of RTs | Same Trait-Different Method | Same Method-Different Trait | Different Method-Different Trait |
| --- | --- | --- | --- |
| Raw RT: Me | .213 | .147 | .254 |
| Ex-Gaussian: tau | .082 | .345 | .030 |
| LNIRT: theta | .038 | .024 | .077 |
| diffIRT: theta | .085 | .030 | .120 |
| Shifted-Wald: Drift | .048 | .073 | .121 |
| Bayesian shifted-Wald: Drift | .113 | .075 | .153 |

**Table S3F:** Spearman's rank correlation coefficients between related AH subtests

| Method | Indicator | CFT2 | CFT3 ratio | E-CSA-WA |
| --- | --- | --- | --- | --- |
| CFT1: Local | Raw RT: Me | .38 [.24, .48] *** | -.01 [-.15, .13] | .31 [.15, .39] *** |
|  | Ex-Gaussian: tau | .03 [-.09, .15] | .03 [-.09, .14] | .02 [-.10, .20] |
|  | LNIRT: theta | -.06 [-.21, .07] | .03 [-.11, .13] | .05 [-.09, .17] |
|  | diffIRT: theta | .11 [-.05, .22] | -.06 [-.18, .04] | .16 [.03, .27] * |
|  | Shifted-Wald: Drift | -.05 [-.19, .08] | -.01 [-.11, .13] | .10 [-.06, .23] |
|  | Bayesian shifted-Wald: Drift | .09 [-.04, .24] | .01 [-.10, .12] | .18 [.05, .29] * |
| CFT1: Global | Raw RT: Me | .33 [.22, .45] *** | .00 [-.14, .12] | .41 [.30, .49] *** |
|  | Ex-Gaussian: tau | .00 [-.14, .11] | .01 [-.13, .12] | .14 [.05, .26] * |
|  | LNIRT: theta | .16 [.04, .31] * | -.03 [-.14, .07] | -.11 [-.22, .01] |
|  | diffIRT: theta | .17 [.07, .28] *** | -.02 [-.15, .20] | .08 [-.03, .20] |
|  | Shifted-Wald: Drift | .10 [-.03, .24] | -.04 [-.16, .09] | .01 [-.12, .14] |
|  | Bayesian shifted-Wald: Drift | .08 [-.07, .23] | -.03 [-.15, .10] | .06 [-.06, .18] |
| CFT2: Local | Raw RT: Me | - | .02 [-.14, .17] | .36 [.20, .47] *** |
|  | Ex-Gaussian: tau | - | .01 [-.14, .17] | .21 [.04, .33] * |
|  | LNIRT: theta | - | .05 [-.08, .19] | .22 [.07, .35] *** |
|  | diffIRT: theta | - | .01 [-.11, .12] | .30 [.16, .43] *** |
|  | Shifted-Wald: Drift | - | -.03 [-.18, .09] | .20 [.06, .33] *** |
|  | Bayesian shifted-Wald: Drift | - | -.07 [-.22, .07] | .26 [.13, .38] *** |
| CFT2: Global | Raw RT: Me | - | -.05 [-.18, .10] | .32 [.14, .42] *** |
|  | Ex-Gaussian: tau | - | .00 [-.15, .16] | .19 [.03, .30] ** |
|  | LNIRT: theta | - | -.06 [-.18, .04] | .25 [.12, .37] *** |
|  | diffIRT: theta | - | -.04 [-.14, .08] | .17 [.05, .30] ** |
|  | Shifted-Wald: Drift | - | .00 [-.12, .13] | .16 [.04, .29] ** |
|  | Bayesian shifted-Wald: Drift | - | .02 [-.12, .16] | .23 [.12, .35] *** |
| E-CSA-WA: Local | Raw RT: Me | - | -.11 [-.23, .03] | - |
|  | Ex-Gaussian: tau | - | -.05 [-.17, .07] | - |
|  | LNIRT: theta | - | -.04 [-.15, .06] | - |
|  | diffIRT: theta | - | -.03 [-.20, .10] | - |
|  | Shifted-Wald: Drift | - | .06 [-.06, .19] | - |
|  | Bayesian shifted-Wald: Drift | - | .05 [-.07, .18] | - |
| E-CSA-WA: Global | Raw RT: Me | - | -.10 [-.23, .04] | - |
|  | Ex-Gaussian: tau | - | -.03 [-.13, .08] | - |
|  | LNIRT: theta | - | -.09 [-.20, .01] | - |
|  | diffIRT: theta | - | .04 [-.08, .15] | - |
|  | Shifted-Wald: Drift | - | .06 [-.05, .17] | - |
|  | Bayesian shifted-Wald: Drift | - | .06 [-.06, .17] | - |

**Table S3G:** Spearman's rank correlation coefficients between subtests within one measure

| Method | Indicator | Associations between subtests |
| --- | --- | --- |
| CFT1 | Raw RT: Me | .83 [.78, .87] *** |
|  | Ex-Gaussian: tau | .03 [-.08, .14] |
|  | LNIRT: theta | -.06 [-.17, .05] |
|  | diffIRT: theta | .15 [.04, .26] ** |
|  | Shifted-Wald: Drift | .16 [.04, .27] * |
|  | Bayesian shifted-Wald: Drift | .20 [.08, .33] ** |
| CFT2 | Raw RT: Me | .89 [.86, .92] *** |
|  | Ex-Gaussian: tau | -.02 [-.12, .09] |
|  | LNIRT: theta | .55 [.48, .62] *** |
|  | diffIRT: theta | .49 [.40, .58] *** |
|  | Shifted-Wald: Drift | .28 [.18, .38] *** |
|  | Bayesian shifted-Wald: Drift | .62 [.52, .68] *** |
| CFT3 | Hierarchical LBA: Drift | -.38 [-.51, -.26] *** |
| E-CSA-WA | Raw RT: Me | .83 [.78, .86] *** |
|  | Ex-Gaussian: tau | .50 [.39, .60] *** |
|  | LNIRT: theta | .55 [.43, .65] *** |
|  | diffIRT: theta | .44 [.35, .54] *** |
|  | Shifted-Wald: Drift | .45 [.32, .54] *** |
|  | Bayesian shifted-Wald: Drift | .56 [.46, .66] *** |

**Table S3H:** Predictive validity of AH methods

| Method | Indicator | Lower mid *M* | Expected direction | Upper mid *M* | *AUC* | 95% *CI* |
| --- | --- | --- | --- | --- | --- | --- |
| CFT1: Local | Raw RT: Me | 1.139 | > | 0.996 | .722 | .612, .831 |
|  | Ex-Gaussian: tau | 0.186 | > | 0.124 | .640 | .514, .766 |
|  | LNIRT: theta | -0.013 | < | -0.029 | .451 | .320, .582 |
|  | diffIRT: theta | 0.991 | < | 1.046 | .538 | ..408, .668 |
|  | Shifted-Wald: Drift | 2.844 | < | 3.289 | .635 | .513, .756 |
|  | Bayesian shifted-Wald: Drift | 4.134 | < | 4.327 | .626 | .500, .753 |
| CFT1: Global | Raw RT: Me | 1.012 | < | 0.874 | .326 | .210, .442 |
|  | Ex-Gaussian: tau | 0.171 | < | 0.177 | .489 | .352, .626 |
|  | LNIRT: theta | -0.003 | > | -0.059 | .426 | .288, .565 |
|  | diffIRT: theta | 0.963 | > | 0.905 | .489 | .347, .631 |
|  | Shifted-Wald: Drift | 2.956 | > | 3.021 | .482 | .342, .622 |
|  | Bayesian shifted-Wald: Drift | 3.602 | > | 3.618 | .489 | .349, .628 |
| CFT2: Local | Raw RT: Me | 2.229 | > | 2.069 | .622 | .469, .776 |
|  | Ex-Gaussian: tau | 0.555 | > | 0.541 | .539 | .372, .705 |
|  | LNIRT: theta | 0.036 | < | 0.080 | .562 | .404, .719 |
|  | diffIRT: theta | 0.948 | < | 1.029 | .565 | .411, .720 |
|  | Shifted-Wald: Drift | 1.712 | < | 1.699 | .469 | .309, .628 |
|  | Bayesian shifted-Wald: Drift | 2.279 | < | 2.357 | .552 | .392, .713 |
| CFT2: Global | Raw RT: Me | 2.244 | < | 2.062 | .367 | .214, .521 |
|  | Ex-Gaussian: tau | 0.515 | < | 0.537 | .519 | .365, .673 |
|  | LNIRT: theta | -0.005 | > | -0.005 | .465 | .307, .623 |
|  | diffIRT: theta | 0.975 | > | 1.043 | .473 | .317, .630 |
|  | Shifted-Wald: Drift | 1.711 | > | 1.743 | .490 | .326, .654 |
|  | Bayesian shifted-Wald: Drift | 2.339 | > | 2.472 | .432 | .271, .594 |
| CFT3: Analytic | Hierarchical LBA: Drift | 0.812 | < | 0.774 | .509 | .367, .651 |
| CFT3: Holistic | Hierarchical LBA: Drift | 2.285 | > | 2.372 | .455 | .320, .590 |
| E-CSA-WA: Local | Raw RT: Me | 1.533 | > | 1.328 | .625 | .498, .751 |
|  | Ex-Gaussian: tau | 0.678 | > | 0.584 | .574 | .441, .706 |
|  | LNIRT: theta | 0.001 | < | -.006 | .440 | .308, .572 |
|  | diffIRT: theta | 1.032 | < | 1.015 | .489 | .355, .623 |
|  | Shifted-Wald: Drift | 1.994 | < | 2.131 | .569 | .437, .701 |
|  | Bayesian shifted-Wald: Drift | 2.681 | < | 2.837 | .576 | .442, .711 |
| E-CSA-WA: Global | Raw RT: Me | 1.293 | < | 1.195 | .435 | .307, .563 |
|  | Ex-Gaussian: tau | 0.460 | < | 0.409 | .404 | .274, .534 |
|  | LNIRT: theta | -0.006 | > | 0.002 | .550 | .419, .682 |
|  | diffIRT: theta | 1.001 | > | 1.048 | .455 | .322, .588 |
|  | Shifted-Wald: Drift | 1.435 | > | 1.568 | .410 | .277, .542 |
|  | Bayesian shifted-Wald: Drift | 2.045 | > | 2.272 | .377 | .247, .507 |
